# Jerveratrum-Type Steroidal Alkaloids Inhibit β-1,6-Glucan Biosynthesis in Fungal Cell Walls

**DOI:** 10.1128/spectrum.00873-21

**Published:** 2022-01-12

**Authors:** Karen Kubo, Kaori Itto-Nakama, Shinsuke Ohnuki, Yoko Yashiroda, Sheena C. Li, Hiromi Kimura, Yumi Kawamura, Yasuhiro Shimamoto, Ken-Ichi Tominaga, Daisuke Yamanaka, Yoshiyuki Adachi, Shinichiro Takashima, Yoichi Noda, Charles Boone, Yoshikazu Ohya

**Affiliations:** a Department of Integrated Biosciences, Graduate School of Frontier Sciences, The University of Tokyo, Kashiwa, Chiba, Japan; b AIST-UTokyo Advanced Operando-Measurement Technology Open Innovation Laboratory (OPERANDO-OIL), National Institute of Advanced Industrial Science and Technology (AIST), Kashiwa, Chiba, Japan; c Research & Development Division, SDS Biotech, Midorigahara, Tsukuba, Ibaraki, Japan; d RIKEN Center for Sustainable Resource Science, Wako, Saitama, Japan; e Terrence Donnelly Centre for Cellular and Biomolecular Research, University of Toronto, Toronto, Ontario, Canada; f Interdisciplinary Research Center for Catalytic Chemistry, National Institute of Advanced Industrial Science and Technology (AIST), Tsukuba, Ibaraki, Japan; g Laboratory for Immunopharmacology of Microbial Products, School of Pharmacy, Tokyo University of Pharmacy and Life Sciences, Hachioji, Tokyo, Japan; h Graduate School of Agricultural and Life Sciences, The University of Tokyo, Bunkyo-ku, Tokyo, Japan; i Collaborative Research Institute for Innovative Microbiology, The University of Tokyo, Bunkyo-ku, Tokyo, Japan; University of Molise

**Keywords:** jervine, antifungal, β-1, 6-glucan, Kre6, Skn1, *Candida*

## Abstract

The limited number of available effective agents necessitates the development of new antifungals. We report that jervine, a jerveratrum-type steroidal alkaloid isolated from Veratrum californicum, has antifungal activity. Phenotypic comparisons of cell wall mutants, K1 killer toxin susceptibility testing, and quantification of cell wall components revealed that β-1,6-glucan biosynthesis was significantly inhibited by jervine. Temperature-sensitive mutants defective in essential genes involved in β-1,6-glucan biosynthesis, including *BIG1*, *KEG1*, *KRE5*, *KRE9*, and *ROT1*, were hypersensitive to jervine. In contrast, point mutations in *KRE6* or its paralog *SKN1* produced jervine resistance, suggesting that jervine targets Kre6 and Skn1. Jervine exhibited broad-spectrum antifungal activity and was effective against human-pathogenic fungi, including Candida parapsilosis and Candida krusei. It was also effective against phytopathogenic fungi, including Botrytis cinerea and Puccinia recondita. Jervine exerted a synergistic effect with fluconazole. Therefore, jervine, a jerveratrum-type steroidal alkaloid used in pharmaceutical products, represents a new class of antifungals active against mycoses and plant-pathogenic fungi.

**IMPORTANCE** Non-Candida albicans *Candida* species (NCAC) are on the rise as a cause of mycosis. Many antifungal drugs are less effective against NCAC, limiting the available therapeutic agents. Here, we report that jervine, a jerveratrum-type steroidal alkaloid, is effective against NCAC and phytopathogenic fungi. Jervine acts on Kre6 and Skn1, which are involved in β-1,6-glucan biosynthesis. The skeleton of jerveratrum-type steroidal alkaloids has been well studied, and more recently, their anticancer properties have been investigated. Therefore, jerveratrum-type alkaloids could potentially be applied as treatments for fungal infections and cancer.

## INTRODUCTION

Fungal infections in humans are primarily caused by Candida, Aspergillus, and Cryptococcus, and they affect millions of people worldwide (1). Mycoses are particularly dangerous for patients with immune systems weakened by cancer, infection with human immunodeficiency virus, or treatment with immunosuppressive drugs (2). However, only four types of antifungal agents are currently used clinically against these fungal species: azoles, polyenes, pyrimidines, and echinocandins. Plant diseases are caused by diverse phytopathogenic fungi and affect a wide range of crops, such as wheat, rice, pepper, rapeseed, potatoes, soybeans, and fruits (3, 4), reducing crop yield and quality and resulting in enormous economic losses (3, 5). The emergence of resistant strains is a major problem in both mycoses and plant diseases (6–9). Therefore, fungal infections pose a serious threat to public health, and new and effective antifungal drugs are needed.

Among the antifungal agents currently used for mycoses, polyenes and azoles, pyrimidines, and echinocandins bind or block the synthesis of ergosterol, disrupt DNA/RNA function, and block β-1,3-glucan biosynthesis, respectively (10). Drug development tends to focus on echinocandins, which act on the fungal cell wall (10, 11), are essential for fungal growth, and are absent from human cells (12, 13). The fungal cell wall is mainly composed of β-1,3-glucan, β-1,6-glucan, chitin, and mannoprotein (10, 14). Drug discovery studies have focused on β-1,3-glucan biosynthesis (10, 11). The echinocandins, including echinocandin B (EB), caspofungin, micafungin, and anidulafungin, bind to and inhibit the catalytic subunit Fks1/2 of β-1,3-glucan synthase (10, 11, 15, 16). In addition, the chitin biosynthesis inhibitor polyoxin B and the cellulose biosynthesis inhibitor 2,6-dichlorobenzonitrile have been proposed for use as fungicides (17). The relatively new antifungal candidate D75-4590 (D75) inhibits β-1,6-glucan biosynthesis (18), providing another means of targeting the fungal cell wall.

The budding yeast Saccharomyces cerevisiae has cell wall components similar to those of human-pathogenic fungi such as Candida and Aspergillus and constitutes a powerful model system for developing new antifungal agents. We previously used chemical reagents and S. cerevisiae to discover a novel antifungal agent derived from plant lignocellulose, named poacic acid (19). We profiled 13,524 compounds using genomic techniques and predicted that some compounds target cell wall biosynthesis and assembly. Further analysis of the morphology and wall components of chemical-treated cells revealed that eight compounds affected the cell wall constituents (20). Four of these compounds (NP157, NP293, NP329, and NP413) are pseudojervines with a skeleton of jerveratrum-type steroidal alkaloids. Jervine was isolated from Veratrum californicum in 1943 (21), and it has been studied as an anticancer agent together with another jerveratrum-type steroidal alkaloid, cyclopamine (22–25). The name “cyclopamine” is derived from “cyclops,” and this alkaloid is a cause of the developmental defect cyclopia (26). Jervine affects yeast cell wall biosynthesis (20); however, it is not yet clear how this bioactive compound functions at the molecular level.

An analysis of cell wall components revealed that jervine inhibits the biosynthesis of β-1,6-glucan. Drug susceptibility tests using mutant strains affecting β-1,6-glucan biosynthesis showed that jervine acts on both Kre6 and Skn1. Also, we found that jervine was effective against human and phytopathogenic fungi. Thus, jerveratrum-type steroidal alkaloids represent a new class of antifungals.

## RESULTS

### Effect of jervine on the yeast cell wall.

**FIG 1:**
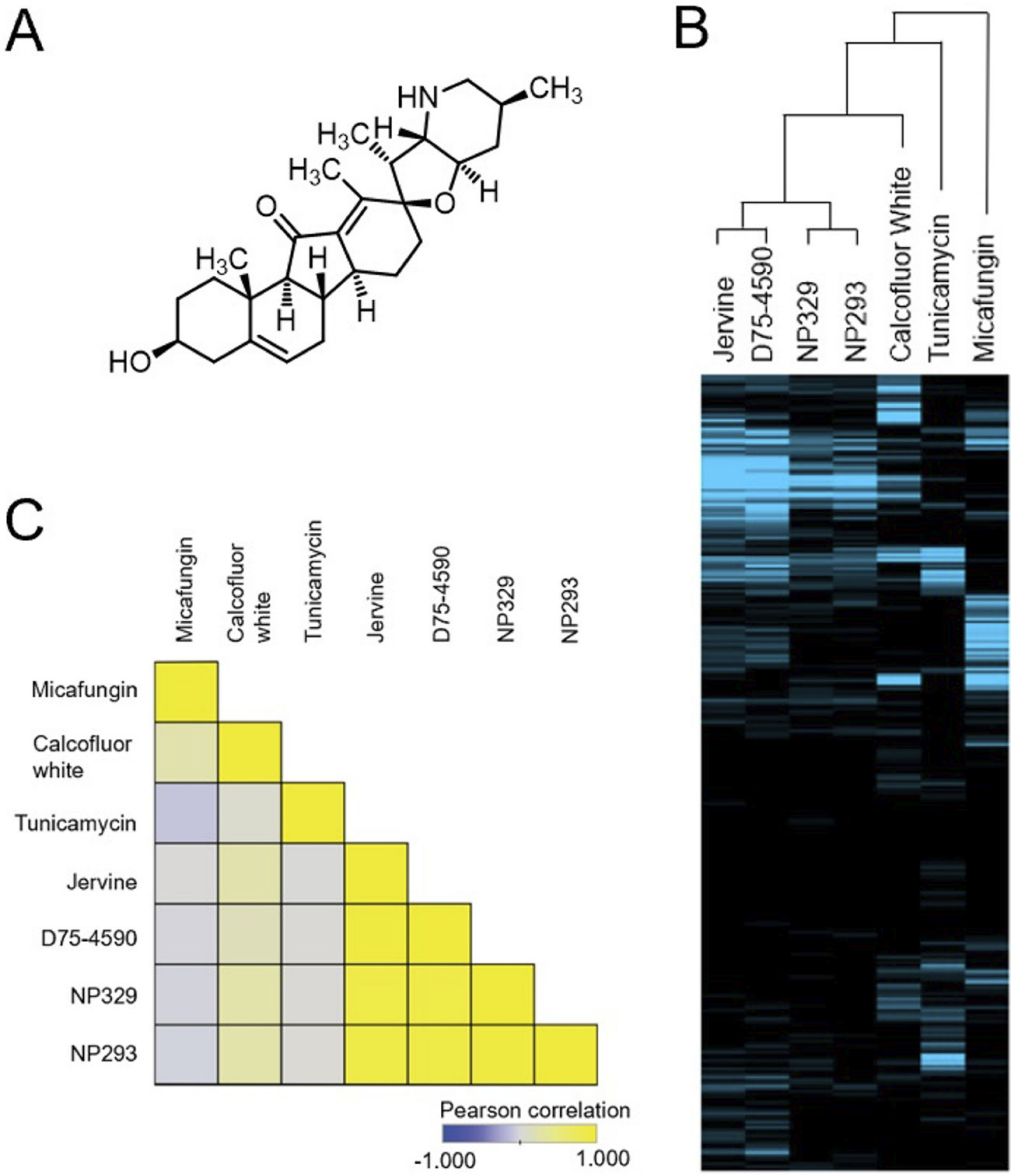
Chemical-genomics profiling after treatment with jervine. (A) Chemical structure of jervine. (B) Chemical-genetic profiles of jervine, D75-4590, NP329, NP293, calcofluor white, and tunicamycin. Chemical-genetic screening was conducted through a nonessential deletion collection of S. cerevisiae mutants. Barcode sequencing data were quantified using BEAN-counter software. Hierarchical clustering was done on both compounds and genes and visualized using Java TreeView with the contrast level set to 5. Blue, negative chemical-genetic interactions, indicating hypersensitivity of the mutants to a compound. (C) Pearson correlation matrix showing the similarities between chemical-genetic profiles. Pearson correlation coefficients (PCC) were calculated for the chemical-genetic profiles based only on negative chemical-genetic interactions. The PCC between two compounds is depicted by a square at the intersection of the compounds. Values closer to 1 are yellow, while those closer to −1 are blue. The chemical-genetic profiles of jervine, D75-4590, NP329, and NP293 are very similar (PCC > 0.8).

We predicted by chemical-genomic analysis that the intracellular targets of pseudojervines are involved in cell wall construction (20). Further phenotypic analysis indicated that jervine (Fig. 1A) and pseudojervines induce cell wall phenotypes (20). Therefore, we compared the chemical-genetic profiles of jervine and pseudojervines with those of other cell wall agents using a functional pool of 310 haploid gene deletion mutants. There was a significant correlation between the chemical-genetic profile of jervine and those of the pseudojervines NP329 and NP293 (Pearson’s correlation coefficient *R* > 0.8, *P* < 0.05 after Bonferroni correction, noncorrelation test) and between those of jervine and D75 (Pearson’s correlation coefficient *R* = 0.93, *P* < 0.05 after Bonferroni correction, noncorrelation test) (Fig. 1B and C). The correlations of jervine with micafungin, calcofluor white, and tunicamycin (TN) were not significant (Fig. 1B and C). Thus, the chemical-genetic profile of jervine is most similar to the chemical-genetic profile of D75, a specific β-1,6-glucan biosynthesis inhibitor (18).

**FIG 2:**
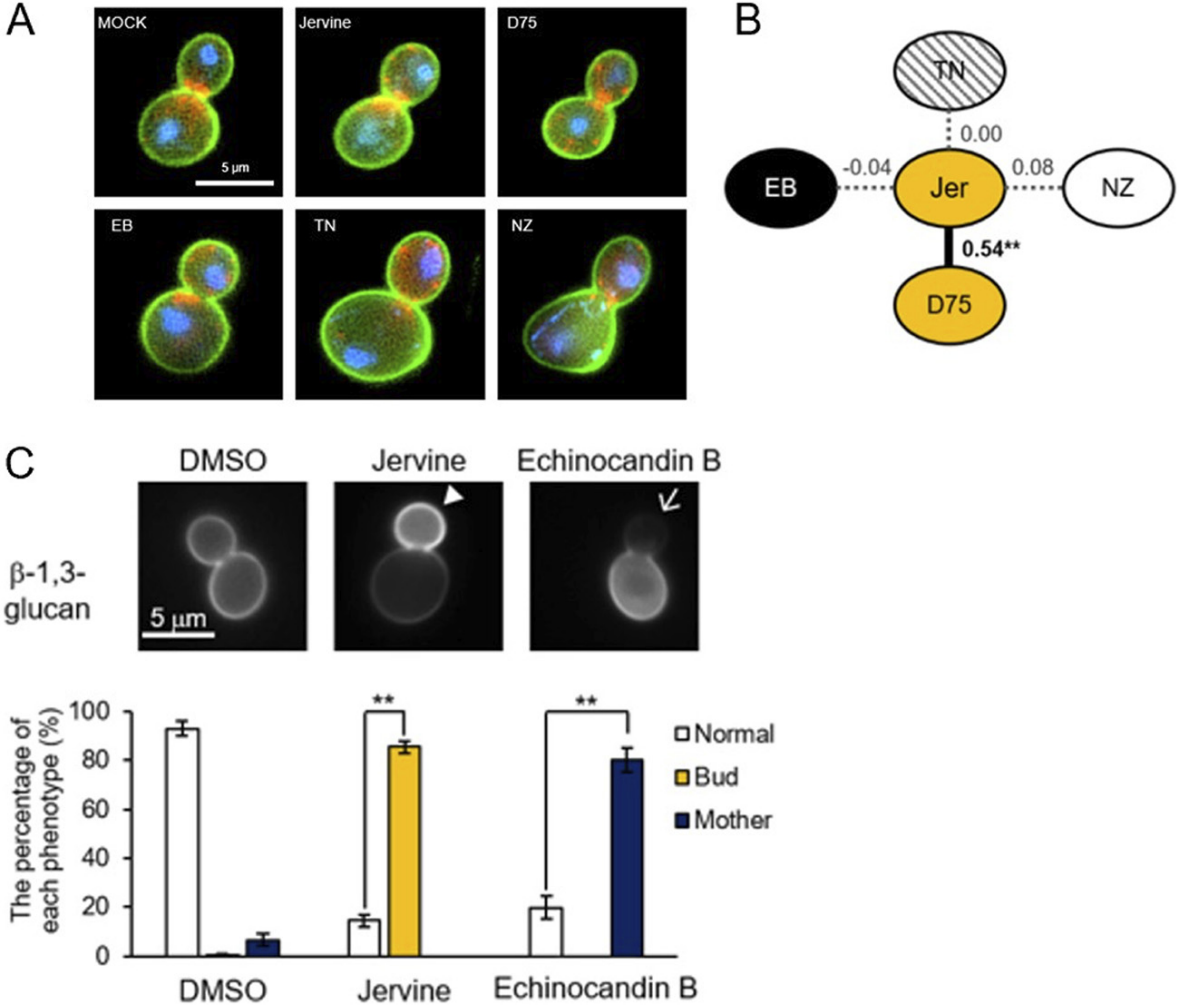
Morphological profiling and localization of β-1,3-glucans after treatment with jervine. (A) Yeast cells treated with the cell wall agents. Cells (green), actin (red), and nuclear DNA (blue) were stained with FITC-ConA, rhodamine-phalloidin, and 4,6-diamidino-2-phenylindole, respectively. EB, echinocandin B (black); TN, tunicamycin (hatching); NZ, nikkomycin Z (white); D75, D75-4590 (gold). (B) Morphological profiling. Nodes and lines indicate drugs and Pearson’s rank correlation *R* between morphological profiles of drug-treated cells. **, *P* < 0.01 after Bonferroni correction, noncorrelation test. (C) Staining of β-1,3-glucan with aniline blue. Wild-type cells were cultured with 1% DMSO, 10 μg/ml jervine, or 4 μg/ml EB for 2 h and stained with aniline blue. The arrowhead (or arrow) indicates increased (or decreased) β-1,3-glucan. More than 150 budded cells were observed and quantified according to three phenotypes: β-1,3-glucan intensity is identical in bud and mother cells (normal), the intensity of bud cells is higher than that of mother cells (bud), and the intensity of mother cells is higher than that of bud cells (mother). **, *P* < 0.01 after Bonferroni correction, *t* test.

Next, we compared the morphological phenotypes of yeast cells treated with the cell wall agents after quantifying cell, actin, and nuclear morphology using CalMorph (27). Jervine had effects on yeast cell morphology similar to those of D75 (Pearson’s rank correlation *R* = 0.54, *P* < 0.01 after Bonferroni correction, noncorrelation test) but not to those of EB, TN, or nikkomycin Z (NZ) (Fig. 2A and B). Treatment with jervine or D75 resulted in slightly smaller cells with a wider neck (Fig. 2A). Cells treated with jervine and EB showed different β-1,3-glucan staining patterns (Fig. 2C). EB-treated cells had weak β-1,3-glucan staining in buds due to decreased β-1,3-glucan biosynthesis. In contrast, jervine-treated cells had strong β-1,3-glucan staining in buds (*P* < 0.01 after Bonferroni correction, *t* test) (Fig. 2C), probably due to a compensatory mechanism. Like jervine, cyclopamine and D75 also showed strong β-1,3-glucan staining in buds, whereas TN and NZ had little effect on β-1,3-glucan staining (Fig. S1A in the supplemental material). Therefore, jervine has effects on yeast cells similar to the effects of D75 but different from those of other cell wall-targeting agents, such as EB, TN, and NZ.

### Jervine inhibits β-1,6-glucan biosynthesis.

**FIG 3:**
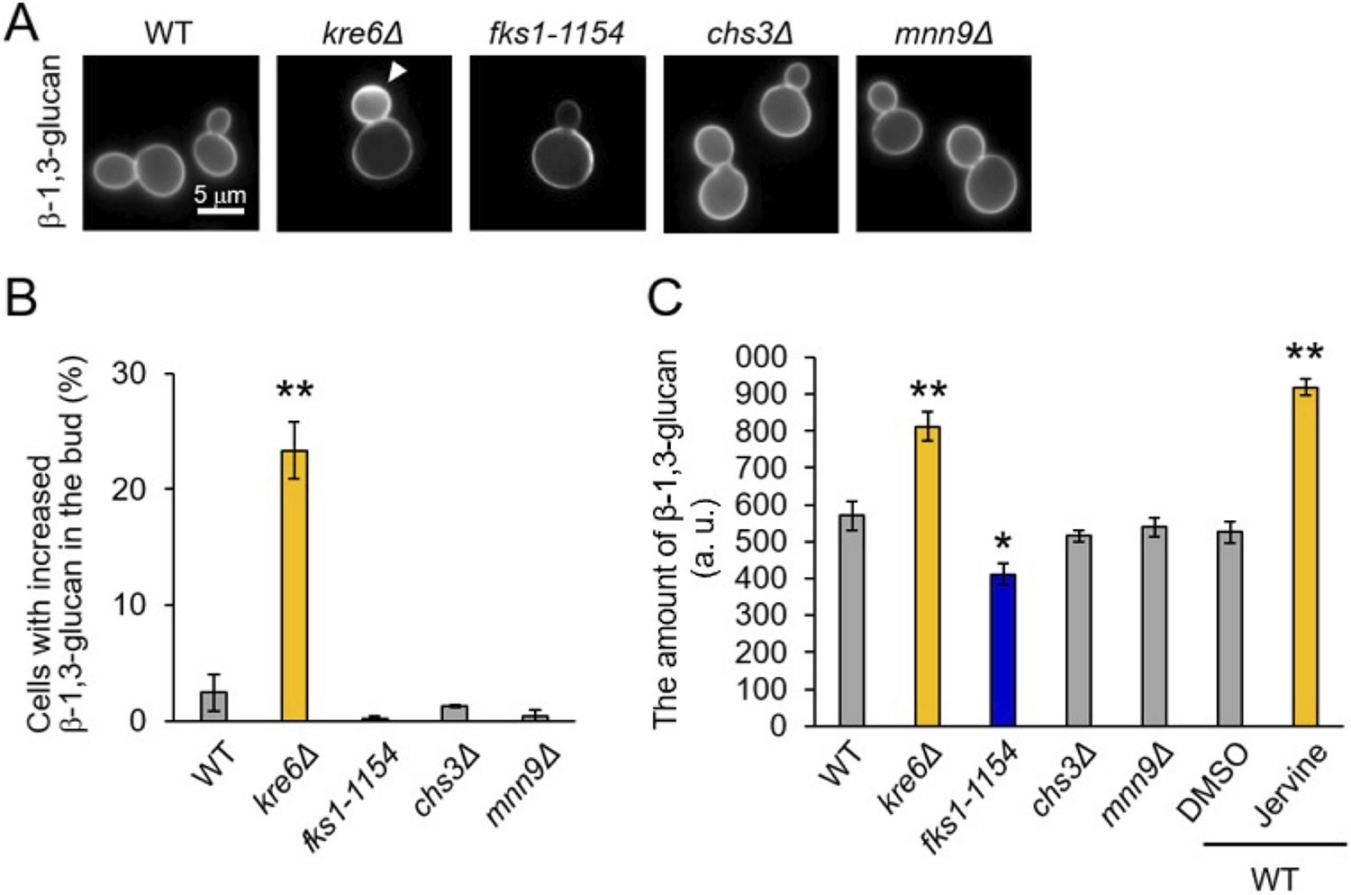
β-1,3-Glucan staining phenotype of yeast mutants defective in the cell wall synthesis pathway. (A) Aniline blue staining of the wild type (WT), β-1,6-glucan synthase mutant (*kre6Δ*), β-1,3-glucan synthase mutant (*fks1-1154*), chitin synthase mutant (*chs3Δ*), and mannosyltransferase mutant (*mnn9Δ*). The yeast strains were cultured to log phase in YPD medium at 25°C and stained with aniline blue. Because the *fks1-1154* strain is a temperature-sensitive mutant (TS mutant), it was cultured overnight at 25°C and cultured for 4 h at restricted temperature (37°C). Arrowhead indicates accumulation of β-1,3-glucan in the bud. (B) Cells with increased β-1,3-glucan in the bud. More than 150 budded cells were observed; the proportions of cells exhibiting β-1,3-glucan accumulation in the bud are shown. Error bars indicate standard deviations. Significant differences from the wild-type strain are indicated with asterisks (**, *P* < 0.01 after Bonferroni correction, *t* test). (C) β-1,3-Glucan contents of the cell wall. More than 150 cells were observed, and β-1,3-glucan contents were quantified using ImageJ (*n* = 3; a.u., arbitrary units). Error bars indicate standard deviations. Significant differences from the wild-type strain are indicated (*, *P* < 0.05, and **, *P* < 0.01, after Bonferroni correction, *t* test).

Because jervine and D75 had similar effects on yeast cells, the link between jervine and β-1,6-glucan biosynthesis was investigated. As in jervine-treated cells, β-1,3-glucan accumulated significantly in the buds of cells with a deletion of *KRE6*, which is involved in β-1,6-glucan biosynthesis (*P* < 0.01 after Bonferroni correction, *t* test) (Fig. 3A and B). Similar phenotypes were not observed in the β-1,3-glucan synthase (*fks1-1154*), chitin (*chs3Δ*), and mannoprotein (*mnn9Δ*) mutants. Also, jervine-treated cells and *kre6Δ* cells had significantly increased β-1,3-glucan contents (*P* < 0.01 after Bonferroni correction, *t* test) (Fig. 3C). Thus, the β-1,3-glucan phenotypes of jervine-treated cells were the most similar to that of the *kre6Δ* mutant among the cell wall-related mutants tested.

**FIG 4:**
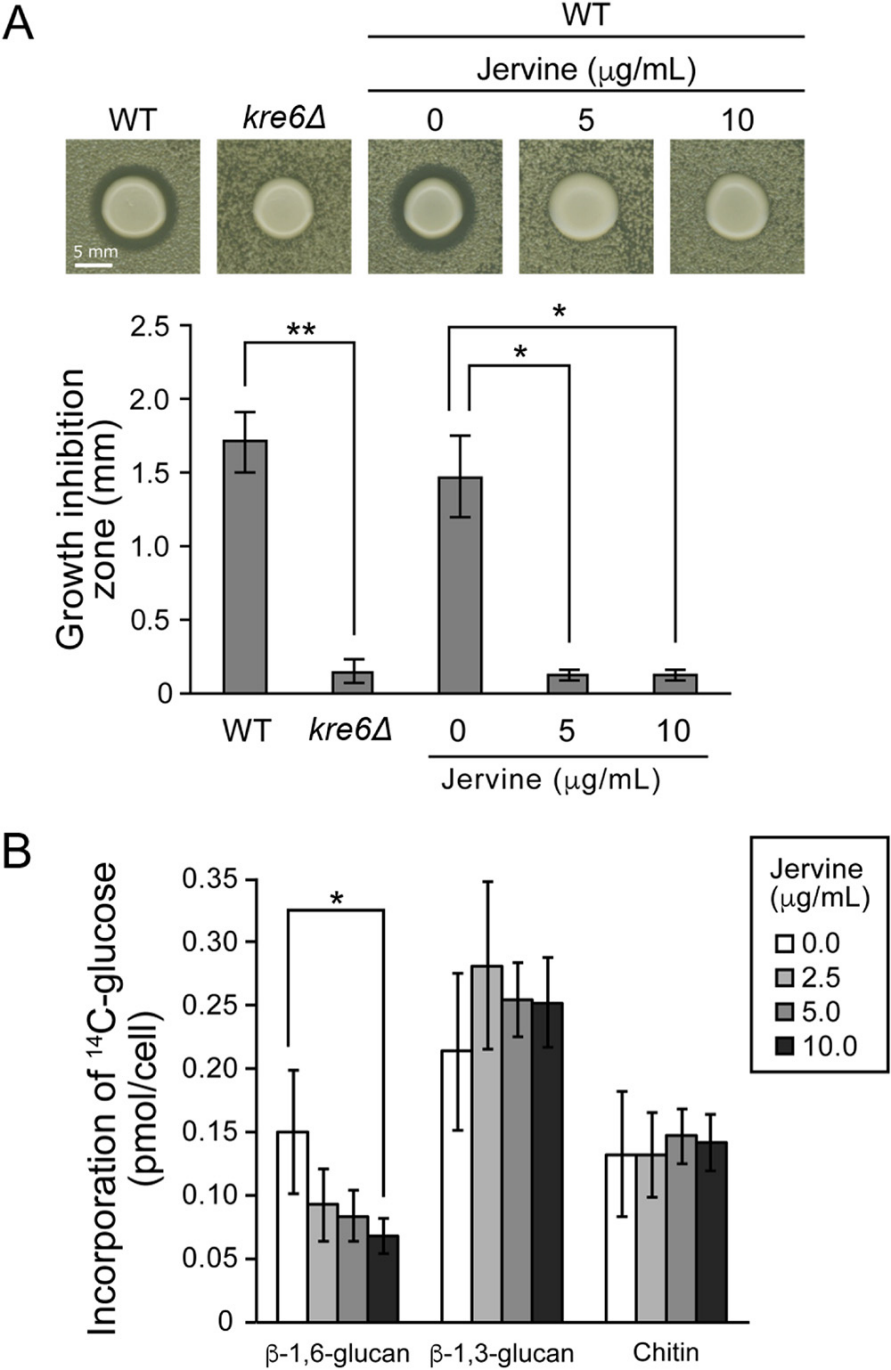
Effect of jervine on β-1,6-glucan biosynthesis. (A) K1 killer toxin susceptibility test. Wild-type (WT) or *kre*6*Δ* cells were added to low-pH YPD as test strains, and the K1 killer toxin-producing strain was spotted at the center. The effect of jervine on the wild-type strain was tested with the presence of 5 or 10 μg/ml of jervine. The large central colony and surrounding area represent the K1 killer toxin-producing strain and test strains, respectively. Growth inhibition zones were quantified with ImageJ (*n* = 3). Error bars indicate standard deviations. Significant differences from the wild-type strain without jervine are indicated (*, *P* < 0.05, and **, *P* < 0.01, after Bonferroni correction, *t* test). (B) Uptake of [^14^C]glucose into cell wall fractions. Wild-type yeast cells were cultured in YPD medium to log phase (1 × 10^7^ cells/ml) and incubated for 2 h in low-glucose YPD medium with [^14^C]glucose (23.1 kBq) in the presence of 0, 2.5, 5.0, or 10.0 μg/ml jervine at 25°C. Alkaline, Zymolyase, and ultrafiltration treatments were performed to obtain β-1,6-glucan, β-1,3-glucan, and chitin fractions, and ^14^C in each fraction was quantified using a scintillation counter (*n* = 4). Error bars indicate standard deviations. *, *P* < 0.05 after Bonferroni correction, *t* test.

K1 killer toxin has a two-step mechanism of action. In step 1, K1 killer toxin binds to β-1,6-glucan in the cell wall. In step 2, the toxin disrupts membrane integrity, which leads to cell death (28, 29). Yeast cells having walls with a high β-1,6-glucan content exhibit a large zone of growth inhibition by K1 killer toxin, whereas mutants that are defective in terms of β-1,6-glucan biosynthesis are often resistant to toxin-mediated cell death (30). The *kre6Δ* strain (with markedly reduced β-1,6-glucan content) had no visible growth inhibition zone (*P* < 0.01 after Bonferroni correction, *t* test) (Fig. 4A). The growth inhibition zone was significantly decreased on plates containing 5 and 10 μg/ml of jervine (*P* < 0.05 after Bonferroni correction, *t* test) (Fig. 4A). We also analyzed the incorporation of ^14^C-labeled glucose into β-1,6-glucan, β-1,3-glucan, and chitin fractions after extraction by mild alkaline lysis and Zymolyase treatment (18). Jervine markedly reduced the radioactivity in only the β-1,6-glucan fraction (Fig. 4B). The reduction in radioactive label incorporation was dose dependent—a significant reduction was observed at 10 μg/ml (*P* < 0.05 after Bonferroni correction, *t* test) (Fig. 4B). These results suggest that jervine specifically inhibits β-1,6-glucan biosynthesis in yeast.

### Effects on yeast strains defective in β-1,6-glucan biosynthesis.

**FIG 5:**
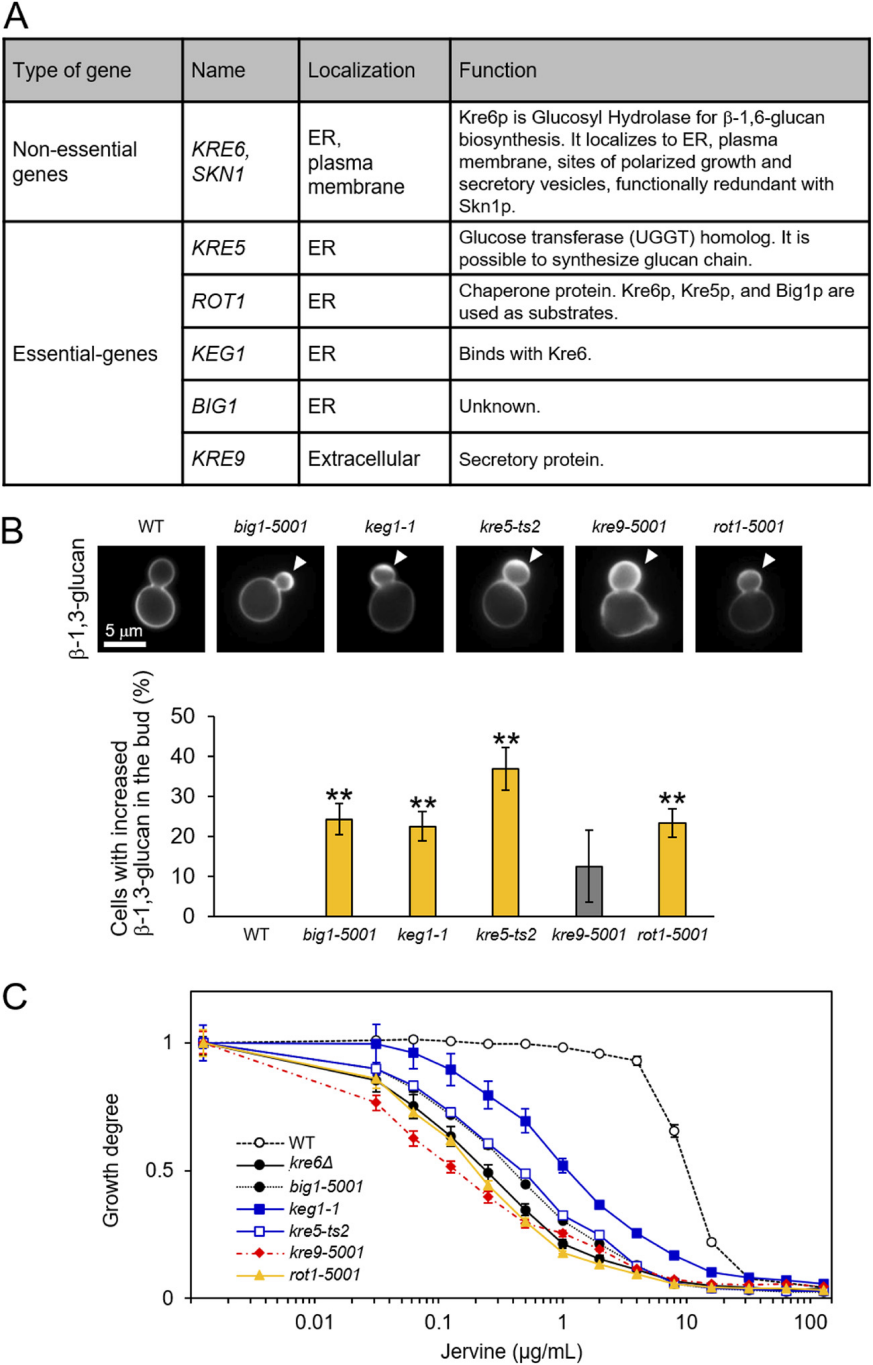
Yeast mutants defective in β-1,6-glucan biosynthesis. (A) Genes involved in β-1,6-glucan biosynthesis. ER, endoplasmic reticulum. (B) Aniline blue staining of mutants defective in β-1,6-glucan biosynthesis. TS mutant cells (*big1-5001*, *keg1-1*, *kre5-ts2*, *kre9-5001*, and *rot1-5001* mutants) were incubated in YPD at 25°C, cultured at 37°C for 6 h, and stained with aniline blue. More than 150 budded cells were observed, and the proportions of cells with accumulated β-1,3-glucan in the bud were counted. Arrowheads indicate accumulation of β-1,3-glucan in the bud. **, *P* < 0.01 after Bonferroni correction, *t* test. (C) Jervine susceptibility test. Wild type (WT) and *kre6Δ* and TS (*kre5-ts2*, *kre9-5001*, *keg1-1*, *big1-5001*, and *rot1-5001*) mutants were cultured in YPD medium supplemented with the indicated concentrations of jervine at 25°C for 18 h. The degree of proliferation was quantitated using the OD_600_; OD_600_ = 1 in the control condition (*n* = 3).

We next examined the effect of jervine on yeast mutant strains defective in β-1,6-glucan biosynthesis. Figure 5A summarizes the genes involved in β-1,6-glucan biosynthesis. Kre6p and Skn1p are putative membrane-associated subunits of related, partially redundant β-1,6-glucan synthases (31). Except for *KRE6* and *SKN1*, the genes involved in β-1,6-glucan biosynthesis are essential, and therefore, we used conditionally lethal, temperature-sensitive (TS) mutants in this analysis. All of the TS mutants examined showed cell wall phenotypes similar to those of *kre6Δ* and jervine-treated cells; the population of cells with accumulated β-1,3-glucan at the buds increased in all TS mutants incubated at the restrictive temperature, with significant increases in the *big1-5001*, *keg1-1*, *kre5-ts2*, and *rot1-5001* strains (*P* < 0.01 after Bonferroni correction, *t* test) (Fig. 5B). These TS mutants were then tested for jervine susceptibility. Compared with the control wild-type strain (half-maximal inhibitory concentration [IC_50_] = 9.7 μg/ml), TS mutant strains defective in β-1,6-glucan biosynthesis exhibited jervine-hypersensitive phenotypes at 25°C. The IC_50_s were 0.1 to 1.0 μg/ml, significantly lower than that of the wild-type strain (likelihood ratio test, *P* < 0.05 after Bonferroni correction) (Fig. 5C and Table 1).

**TABLE 1:** Jervine susceptibilities of yeast strains defective in β-1,6-glucan synthesis^*a*^

Strain description	IC_50_ (μg/mL)	SE
*his3Δ*	9.695	0.171
*kre6Δ*	0.222	0.040
*big1-5001*	0.374	0.020
*keg1-1*	1.077	0.292
*kre5-ts2*	0.432	0.045
*kre9-5001*	0.116	0.032
*rot1-5001*	0.182	0.020

a Strains were grown at 25°C.

### Effects on *KRE6*(*F552I*) and *SKN1*(F604I) mutants.

Because a point mutation in *KRE6* that changed phenylalanine to isoleucine at position 552 [*KRE6*(*F552I*)] induced resistance to D75, *KRE6* was formerly considered the target gene of D75 (18). We report here that the mechanism of action of jervine is similar to that of D75. However, the *kre6Δ* strain showed jervine sensitivity, excluding the possibility that Kre6 is the only target of jervine. *KRE6* has a homolog, *SKN1* (67% amino acid sequence homology), and the *kre6Δ skn1Δ* double deletion mutation is lethal (31). Therefore, to determine whether both Kre6 and Skn1 are targets of jervine, a possible resistance mutation, Phe604Ile, of *SKN1* (corresponding to Phe552Ile of *KRE6*) was examined.

**FIG 6:**
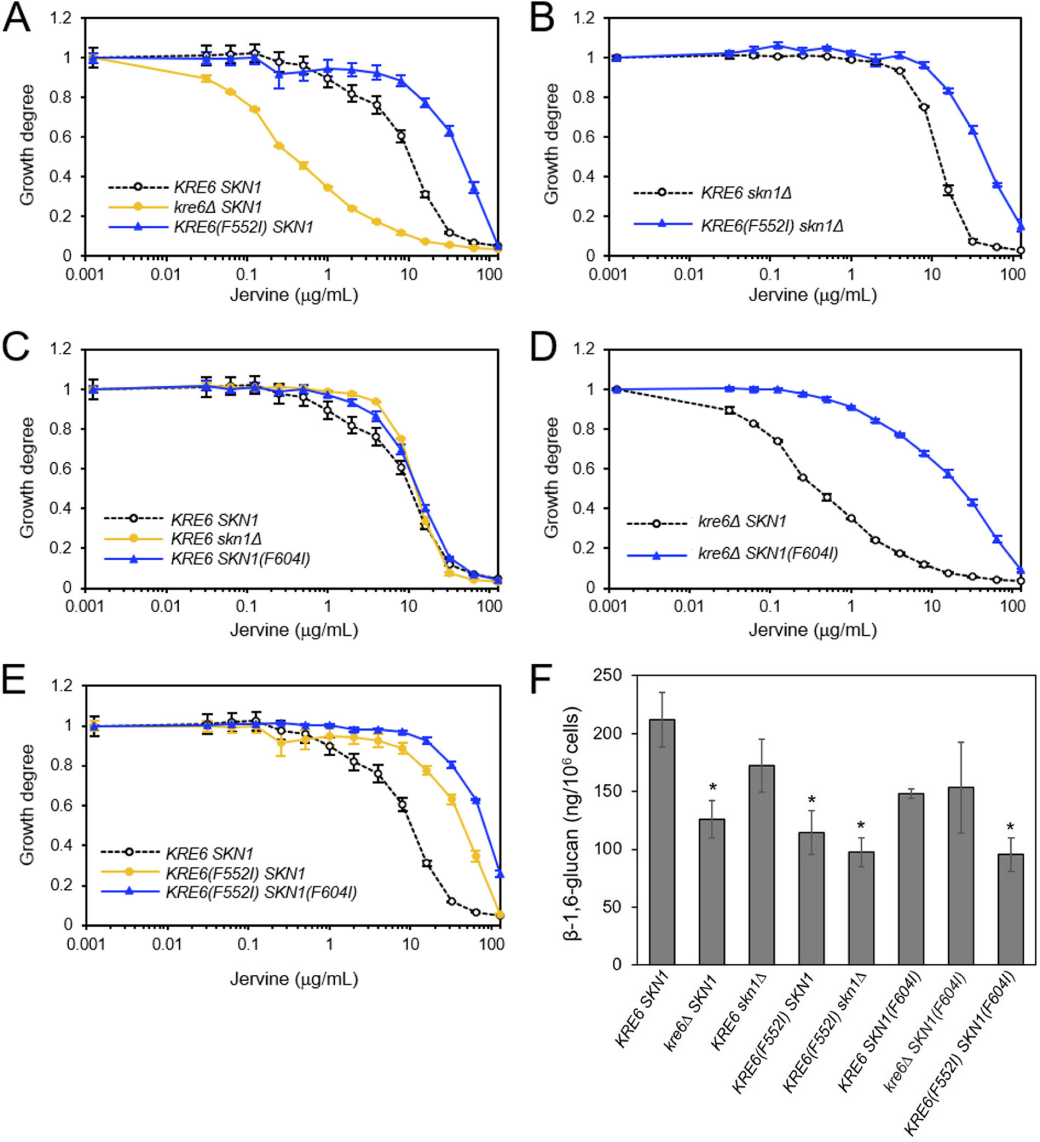
Jervine sensitivity and β-1,6-glucan levels of the wild-type strain and *KRE6*(*F552I*) and *SKN1*(*F604I*) mutants. Yeast mutant strains were incubated in YPD in the presence of the indicated concentrations of jervine at 25°C for 18 h. The degree of proliferation was quantitated using the OD_600_; OD_600_ = 1 in the control condition (*n* = 3). (A) Wild-type (*KRE6 SKN1*) and *kre6Δ SKN1* and *KRE6*(*F552I*) *SKN1* strains. (B) *KRE6 skn1Δ* and *KRE6*(*F552I*) *skn1Δ* strains. (C) *KRE6 SKN1*, *KRE6 skn1Δ*, and *KRE6 SKN1*(*F604I*) strains. (D) *kre6Δ SKN1* and *kre6Δ SKN*(*F604I*) strains. (E) *KRE6 SKN1*, *KRE6*(*F552I*) *SKN1*, and *KRE6*(*F552I*) *SKN1*(*F604I*) strains. (F) β-1,6-Glucan levels in the yeast strains. The amount of glucan per cell was calculated using pustulan as the standard. Significant difference between the wild-type (*KRE6 SKN1*) and mutant strains are indicated (*, *P* < 0.05 after false discovery rate correction, *t* test).

In the *SKN1* background, a *KRE6*(*F552I*) mutant showed significant jervine resistance at 25°C (likelihood ratio test, *P* < 0.05 after Bonferroni correction) (Fig. 6A and Table 2; Fig. S2A), as well as resistance to D75 (Fig. S3A). Even in the *skn1Δ* background, a *KRE6*(*F552I*) mutant showed significant jervine resistance (likelihood ratio test, *P* < 0.05) (Fig. 6B and Table 2). In the *KRE6* background, *skn1Δ* and *SKN1*(*F604I*) mutants showed slight but significant resistance (likelihood ratio test, *P* < 0.05 after Bonferroni correction) (Fig. 6C and Table 2). In addition, in the *kre6*Δ background, a *SKN1*(*F604I*) mutant exhibited significant resistance to jervine (likelihood ratio test, *P* < 0.05) (Fig. 6D and Table 2), clearly indicating that *SKN1*(*F604I*) induced jervine resistance. The *KRE6*(*F552I*) *SKN1*(*F604I*) double mutants had the most jervine-resistant phenotype (Fig. 6E). At 30°C, no jervine resistance caused by *skn1Δ* was observed (Fig. S2B). However, at 30°C, both *KRE6*(*F552I*) and *SKN1*(*F604I*) mutants still showed jervine resistance (Fig. S2B). The D75 susceptibility pattern was identical to that of jervine at both 25°C and 30°C (Fig. S3A and B).

**TABLE 2:** Jervine susceptibilities of the wild-type strain and the *KRE6*(*F552I*), and *SKN1*(*F604I*) mutants^*a*^

Strain description	IC_50_ (μg/mL)	SE
KRE6 SKN1	9.602	0.558
KRE6(F552I) SKN1	101.732	1.006
KRE6 skn1Δ	11.894	0.225
kre6Δ SKN1	0.363	0.019
KRE6 SKN1(F604I)	12.516	0.467
kre6Δ SKN1(F604I)	80.322	0.992
KRE6(F552I) Δskn1	45.134	0.987
KRE6(F552I) SKN1(F604I)	700.225	0.999

a Strains were grown at 25°C.

To investigate whether jervine resistance was acquired via increased β-1,6-glucan production by the mutant proteins Kre6(F552I) and Skn1(F604I), we next examined whether β-1,6-glucan increased in these mutants by using a newly developed β-1,6-glucan detection method, which exploits a specific β-1,6-glucan probe generated by modifying recombinant Neg1, a Neurospora crassa endo-β-1,6-glucanase (32). We found that none of the mutants harboring *KRE6*(*F552I*) or *SKN1*(*F604I*) contained more β-1,6-glucan than the *KRE6 SKN1* strain (Fig. 6F). Rather, *kre6Δ SKN1*, *KRE6*(*F552I*) *SKN1*, *KRE6*(*F552I*) *skn1Δ*, and *KRE6*(*F552I*) *SKN1*(*F604I*) mutants had significantly decreased β-1,6-glucan compared with the control *KRE6 SKN1* strain (*P* < 0.05 after false discovery rate correction, *t* test) (Fig. 6F). This indicated that the acquisition of jervine resistance was not due to increased β-1,6-glucan production.

Next, we tested whether the point mutations conferred protection against jervine in yeast cells. Although treatment with jervine significantly reduced the β-1,6-glucan levels in *KRE6 SKN1* and *KRE6 skn1Δ* cells (*P* < 0.05 after Bonferroni correction, *t* test) (Fig. S4), no significant change in β-1,6-glucan biosynthesis was detected in *KRE6*(*F552I*) *skn1Δ* cells, suggesting that Kre6(F552I) cells were insensitive to jervine. *kre6Δ SKN1*(*F604I*) cells still exhibited some sensitivity to jervine, suggesting that this mutation conferred weak protection against jervine. These results suggest that jervine acts on Kre6 and Skn1, inhibiting β-1,6-glucan biosynthesis.

### Antifungal spectrum.

**FIG 7:**
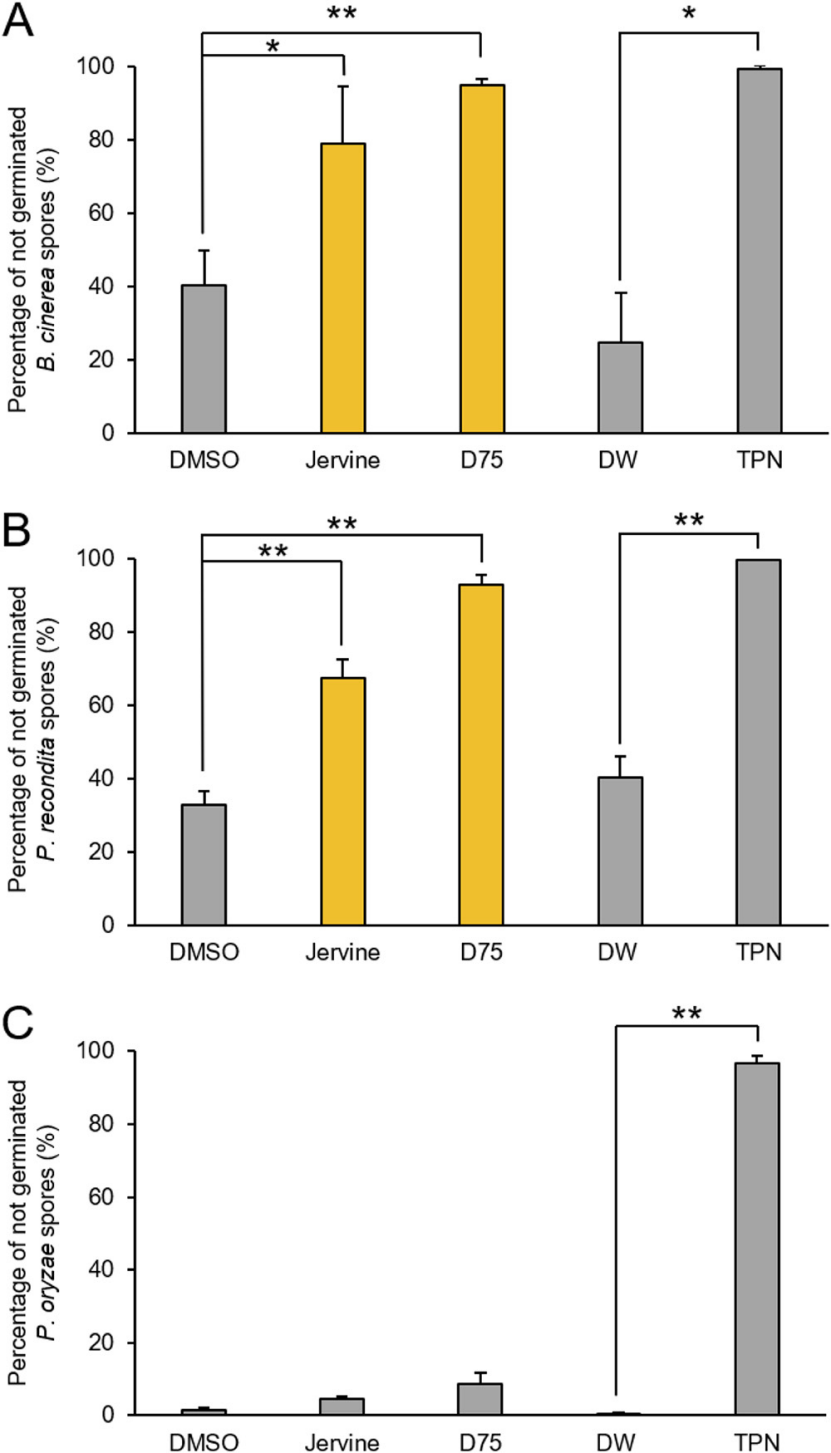
Germination inhibitory activity of jervine against phytopathogenic fungi. Spores of *B. cinerea* (A), *P. recondita* (B), and *P. oryzae* (C) were cultured with 50 μg/ml of jervine or D75 or 10 μg/ml of 2,4,5,6-tetrachloroisophthalonitrile for 48 h. More than 150 spores were examined, and the percentages of nongerminating cells were evaluated (*n* = 3). Error bars indicate standard deviations. *, *P* < 0.05, and **, *P* < 0.01, after Bonferroni correction, *t* test. D75, D75-4590; DW, distilled water; TPN, 2,4,5,6-tetrachloroisophthalonitrile.

The antifungal spectrum of jervine was investigated using human-pathogenic fungi and phytopathogenic fungi. *Candida* species—including Candida albicans, Candida glabrata, Candida tropicalis, Candida parapsilosis, and Candida krusei—are the major causative fungi of invasive human mycoses (2). We found that jervine was highly effective against C. parapsilosis and C. krusei (Table 3). Specifically, jervine was more effective than fluconazole (FLC) against C. krusei and more effective than EB against C. parapsilosis (Table 3). Therefore, jervine can be used as an alternative when FLC or EB is not sufficiently effective. The germination inhibitory effect of jervine on the phytopathogenic fungi Botrytis cinerea, Puccinia recondita, and Pyricularia oryzae was next investigated. Jervine at 50 μg/ml inhibited the germination of B. cinerea significantly, by 80% (*P* < 0.05 after Bonferroni correction, *t* test) (Fig. 7A). Jervine inhibited P. recondita germination by 60% (*P* < 0.01 after Bonferroni correction, *t* test) (Fig. 7B). However, the phytopathogenic fungus P. oryzae, which lacks β-1,6-glucan, was resistant to jervine (Fig. 7C). A similar tendency was observed for D75 (Fig. 7A to C). These results unveiled the antifungal spectrum of jervine against human-pathogenic fungi and phytopathogenic fungi.

**TABLE 3:** Antifungal activities of antifungal agents in CLSI method

Strain	Value for^*a*^:					
	Jervine		FLC		EB	
	MIC_90_	MIC_50_	MIC_90_	MIC_50_	MIC_90_	MIC_50_
S. cerevisiae Δ*his3*	4	≤0.125	4	1–4	4	4
C. albicans ATCC 24433	>64	>64	4	0.125–0.25	1	0.25–0.5
C. parapsilosis ATCC 22019	8	8	1	1	32	32
C. tropicalis ATCC 750	>64	16	8	0.5	1	≤0.125
C. krusei ATCC 6258	8	4	32	32^*b*^	4	4

a MIC was tested using RPMI as the medium by CLSI M60 methods (64) and was determined after 24 or 48 h incubation. MIC_50_ was defined as a prominent decrease in turbidity compared with that of a drug-free control, and MIC_90_ was defined as the lowest drug concentration supporting no visible growth after 24 or 48 h of incubation. FLC, fluconazole; EB, echinocandin B.

b The quality control was verified according to the criteria described in CLSI M60 (61).

### Combination therapy.

**FIG 8:**
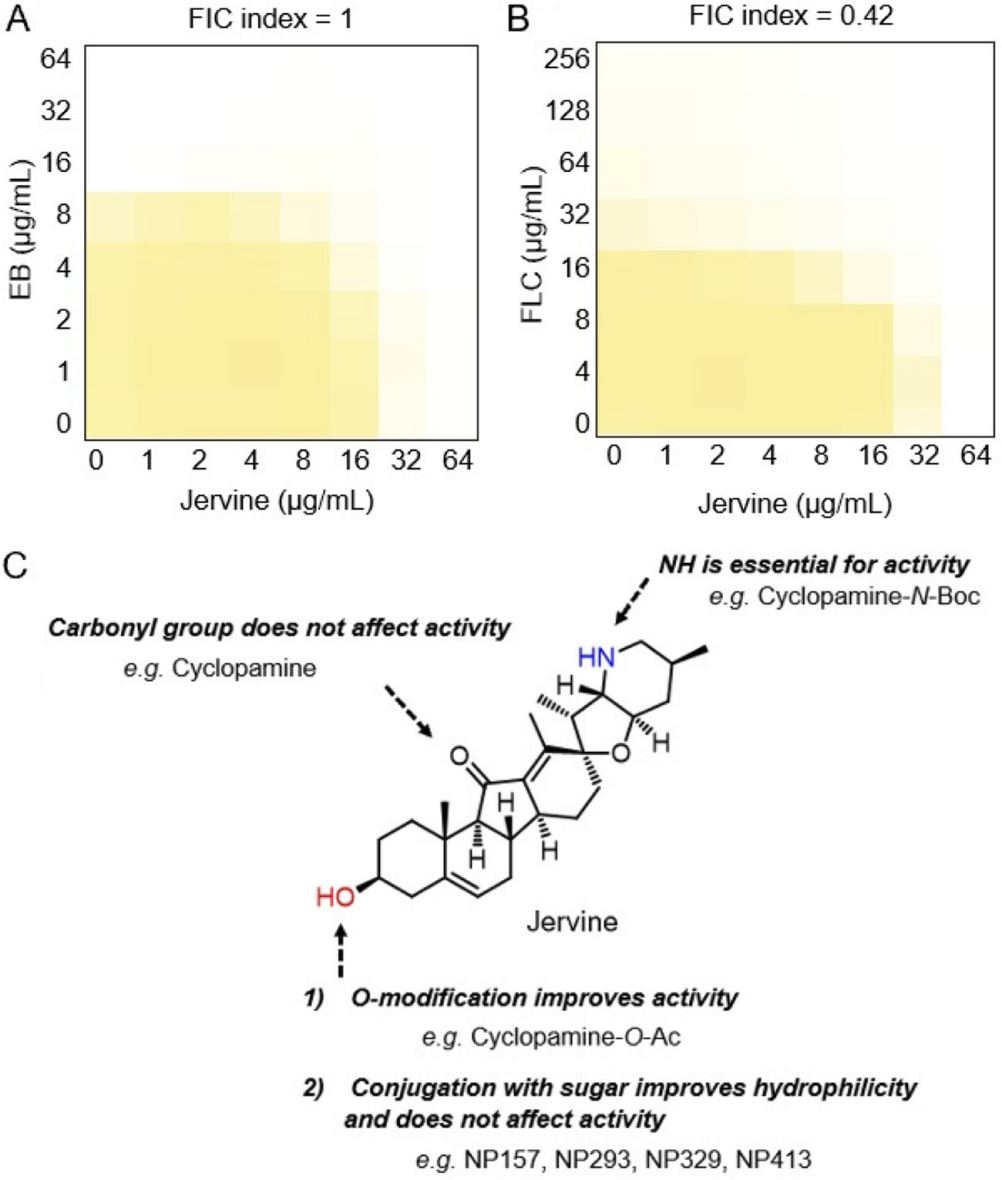
Checkerboard assays of jervine and the structure required for jervine activity. The wild-type strain was incubated in YPD in the presence of 0 to 64 μg/ml EB and 0 to 64 μg/ml jervine (A) or 0 to 256 μg/ml FLC and 0 to 64 μg/ml jervine (B) at 25°C for 18 h. The degree of proliferation was quantitated using the OD_600_; that of the control (2% DMSO) was set as 1 (*n* = 3). Yellow regions represent higher cell densities. The FIC index represents the effect of the combined use of the two compounds and is expressed as the mean value from three biological replicates. (C) Structural moieties essential for the activity of jerveratrum-type steroidal alkaloids.

There are only four types of antifungal agents in clinical use. If a single agent is not effective, combination therapy is attempted (33). The combined use of amphotericin B and flucytosine is effective in cryptococcosis (34). Here, we examined the efficacy of combinations of jervine with EB or FLC in S. cerevisiae. Jervine with EB (Fig. 8A) was effective; more importantly, jervine with FLC showed synergistic effects on the growth rate (fractional inhibitory concentration [FIC] index < 0.5) (Fig. 8B). These results suggest that jervine is effective in combination with EB and FLC.

### Structural moieties essential for activity of jerveratrum-type steroidal alkaloids.

We aimed to identify the steroidal alkaloid residues important for antifungal activity. The IC_50_s of jervine and cyclopamine against S. cerevisiae were similar (Table 4), indicating that the ketone group of jervine is not important for its antifungal activity. The IC_50_ of cyclopamine-*N*-Boc (di-*tert*-butyl dicarbonate) was ∼10-fold that of cyclopamine (*P* < 0.01 after Bonferroni correction, likelihood ratio test) (Table 4). This indicated that the amine in the piperidine skeleton is important for antifungal activity, likely forming hydrogen bonds with target molecules. When NP157, NP293, NP329, and NP413 were compared with jervine, there was little difference (Table 4). This may constitute useful information for studies on structure-activity relationships aiming to improve water solubility (35). Cyclopamine-*O*-Ac (acetyl), in which the same modification site was acetylated in cyclopamine, had a slightly but significantly lower IC_50_ (*P* < 0.01 after Bonferroni correction, likelihood ratio test) (Table 4), demonstrating that the secondary alcohol in the A ring has some effect on antifungal activity. Therefore, modification of this secondary alcohol via ester and carbamate bonds may increase activity. The basic structure common to these jerveratrum-type steroid alkaloids is the 6-6-5-6 ABCD ring system and its spiro-connected aza-bicyclo[4.3.0]nonane ring. Because jervine has an α,β-unsaturated ketone, it may have a more planar CD ring than cyclopamine. In addition, ring expansion modifications of the basic skeleton by construction of 6-6-5-7 and 7-6-5-7 ABCD ring systems enhance cyclopamine activity (36–39). The structure-activity relationships of jerveratrum-type steroid alkaloids (Fig. 8C) will contribute to the design of molecules that are more selective for pathogenic fungi.

**TABLE 4:** IC_50_s of drugs against S. cerevisiae wild type (*his3*Δ)

Drug	IC_50_ ± SD for indicated unit of measure^*a*^		*P* value^*b*^
	μg/mL	μM	
Jervine	5.844 ± 0.594	13.74 ± 1.40	
Cyclopamine	7.238 ± 0.573	17.60 ± 1.39	
Cyclopamine-*N*-Boc	77.183 ± 0.991	150.93 ± 1.94	3.88E−05
Cyclopamine-*O*-Ac	2.705 ± 0.481	5.97 ± 1.06	5.29E−04
NP157	14.279 ± 0.989	24.31 ± 1.68	
NP293	8.975 ± 0.879	15.28 ± 1.50	
NP329	9.524 ± 0.915	16.22 ± 1.56	
NP413	8.235 ± 0.892	14.02 ± 1.52	

a The wild type was cultured in YPD at 25°C for 24 h in the presence of 64, 32, 16, 8, 4, 2, 1, 0.5, and 0 μg/mL of drugs. IC_50_s of each drug are shown in the table. The experiment was repeated with similar results. Values for two different measures of concentration are shown because each compound has a different molecular weight.

b Significant difference from the value for cyclopamine (*P* < 0.01, likelihood ratio test).

## DISCUSSION

The fungal cell wall is a target for antifungals because it has components absent in humans and plants (12, 13). We presented evidence that jerveratrum-type alkaloids are antifungal agents that inhibit fungal cell wall biosynthesis via a mechanism different from those of EB and NZ and affect β-1,6-glucan biosynthesis. Jervine acts on Kre6 and Skn1, both involved in β-1,6-glucan biosynthesis. A combination drug test for human mycosis suggested the antifungal potential of jervine. The skeleton of jerveratrum-type steroidal alkaloids has been studied for over 100 years (40), and more recently, its anticancer potential has been investigated (22–25). Therefore, jerveratrum-type alkaloids have potential as antifungals, linking cancer and antifungal treatment.

### Jervine inhibits β-1,6-glucan biosynthesis.

Evaluation of cell wall phenotypes, K1 killer toxin sensitivities, and cell wall components indicated that jervine inhibits β-1,6-glucan biosynthesis. Furthermore, jervine had less effect on the phytopathogenic fungus P. oryzae, which lacks β-1,6-glucan. For β-1,6-glucan biosynthesis, *KRE6* and *SKN1* are essential (31, 41, 42). The double gene deletion mutation is lethal and causes deficiency of cell division, abnormal cell walls, decreased hyphal growth, decreased biofilm formation, and loss of pathogenicity in mice (31). The *KRE6*(*F552I*) or the *SKN1*(*F604I*) mutation resulted in jervine resistance. A single deletion mutant with an *skn1Δ* mutation also showed a weak jervine-resistant phenotype. In addition, the *kre5-ts2*, *rot1-5001*, *keg1-1*, *big1-5001*, and *kre9-5001* mutant strains defective in β-1,6-glucan biosynthesis were all sensitive to jervine. These lines of genetic evidence suggest that jervine binds directly to Kre6 and Skn1 to block β-1,6-glucan biosynthesis.

### Mechanism of β-1,6-glucan biosynthesis.

Although many factors involved in β-1,6-glucan biosynthesis have been identified, its biosynthesis remains unclear. One reason is delayed development of biochemical technology. For example, aniline blue stains β-1,3-glucan, but there was no stain specific for β-1,6-glucan and its intermediate products. From this perspective, the recent development of β-1,6-glucan-specific probes is promising (32). Kinetic studies of β-1,3-glucan biosynthesis have been conducted by tracing the formation of glucose chains (43), but the biosynthesis of β-1,6-glucan chains is unclear. EB likely binds directly to the catalytic subunit Fks1 of β-1,3-glucan synthase (44, 45), but the catalytic subunit of β-1,6-glucan synthase has not been identified. Because jervine acted on Kre6 and Skn1, we plan to study the involvement of Kre6 and Skn1 in β-1,6-glucan biosynthesis.

### Comparison of jervine and D75.

Although jervine and D75 (18) and its derivatives (D11-2040 and D21-6076) (46, 47) have different chemical structures, their antifungal activities have the same mechanism. This implies that β-1,6-glucan biosynthesis inhibitors have at least two types of maternal skeleton (jervine, steroid skeleton; D75, heterocyclic skeleton). The development of new antifungals that target the cell wall is desired. As a result, β-1,3-glucan biosynthesis inhibitors have been developed, but only one type of echinocandin is in use (10). Because there is only one echinocandin-based maternal skeleton (cyclic peptide) and various echinocandin-based derivatives were developed by comprehensive chemical synthesis targeting side chains (10), the echinocandin-based derivatives may not be effective against fungi that have acquired resistance to echinocandin itself. In contrast, β-1,6-glucan biosynthesis inhibitors have at least two types of chemical structure. Drug discovery research on D75 has been conducted (46, 47), and jervine, with its significantly different maternal skeleton, will likely be effective against D75-resistant fungi. Simultaneous development of jervine and D75 may lead to the discovery of additional antifungal agents.

### Jervine is effective against NCAC.

Non-Candida albicans *Candida* species (NCAC) are on the rise as a cause of mycosis (48–50). Many antifungal drugs are generally considered to be less effective against NCAC (51–53), so the available therapeutic agents are limited (52). NCAC include C. parapsilosis and C. krusei, the growth of which jervine inhibited *in vitro* at low concentrations. Candida parapsilosis has marked biofilm-forming ability (2) and, as a result, is less susceptible to echinocandin-based derivatives (54), implying that our findings are of importance. D75 is not effective against C. parapsilosis (18). Therefore, jervine is the only alternative drug for C. parapsilosis mycosis when FLC is not sufficiently effective. It should be noted that jervine is more selective toward fungal than human cells; jervine concentrations higher than 160 μg/ml are required for the inhibition of nontumor epithelial cells (25). Jervine is not recommended for use in humans due to the teratogenic potential of jerveratrum-type steroidal alkaloids (55). However, jervine could be explored as a scaffold for the development of novel antifungal agents in the future.

### Conclusion.

In this study, we demonstrated the antifungal activity of jerveratrum-type steroidal alkaloids, such as jervine and cyclopamine, for the first time. These compounds inhibited the growth of the human-pathogenic fungi C. parapsilosis and C. krusei, as well as the phytopathogenic fungi B. cinerea and P. recondita. Jervine did not impact the growth of P. oryzae, which lacks β-1,6-glucan. We found that jervine significantly inhibits β-1,6-glucan biosynthesis in S. cerevisiae. Jervine acts on Kre6 and Skn1, which are involved in β-1,6-glucan biosynthesis. Furthermore, jervine acts synergistically with fluconazole. These findings support the future development of jerveratrum-type steroidal alkaloids as antifungal agents.

## MATERIALS AND METHODS

### Strains.

An S. cerevisiae *his3Δ*(*MAT***a** *his3*::*kanMX4 leu2 met15 ura3*) strain, a derivative of BY4741 harboring a *kanMX4* cassette at the *his3* locus, was used as the wild-type strain unless otherwise indicated. Various *MAT***a** haploid gene deletion strains (with deletions of *his3Δ*, *kre6Δ*, *chs3Δ*, *mnn9Δ*, and *skn1Δ*) were obtained from the European Saccharomyces cerevisiae Archive for Functional Analysis (Euroscarf). The *Candida* strains (C. albicans ATCC 24433, C. krusei ATCC 6258, C. tropicalis ATCC 750, and C. parapsilosis ATCC 22019) were obtained from National BioResource Project Pathogenic eukaryotic microorganisms. The other yeast strains used are listed in Table 5.

**TABLE 5:** Fungal strains used in this study

Species	Strain		Genotype^*a*^	Reference(s) or source
	YOC no.	Alias or mutation(s)		
S. cerevisiae	YOC5130	Y13206	*MAT***a** *pdr1*::*natMX pdr3*::*Kl.URA3 snq2*::*Kl.LEU2 can1*::*STE2*pr*-Sp.his5 his3 lue2 lyp1 met15 ura3*^*b*^	20
	YOC4002	BY4741	*MAT***a** *his3 leu2 met15 ura3*	Euroscarf Collection
		*his3Δ* (wild type)	*MAT***a** *his3*::*kanMX4 leu2 met15 ura3*	Euroscarf Collection
		*kre6Δ*	*MAT***a** *his3 leu2 met15 ura3 kre6*::*kanMX4*	Euroscarf Collection
		*chs3Δ*	*MAT***a** *his3 leu2 met15 ura3 chs3*::*kanMX4*	Euroscarf Collection
		*mnn10Δ*	*MAT***a** *his3 leu2 met15 ura3 mnn10*::*kanMX4*	Euroscarf Collection
		*rot1-5001*	*MAT***a** *his3 leu2 met15 ura3 rot1-5001*:*kanMX*	65
		*kre5-ts2*	*MAT***a** *his3 leu2 met15 ura3 kre5-ts2*:*kanMX*	65
		*kre9-5001*	*MAT***a** *his3 leu2 met15 ura3 kre9-5001*:*kanMX*	65
		*big1-5001*	*MAT***a** *his3 leu2 met15 ura3 big1-5001*:*kanMX*	65
	YOC5443	*keg1-1*	*MAT***a** *his3 leu2 trp1 ura3 keg1-1*:*TRP1*	66
	YOC1087	*fks1-1154*	*MAT***a** *ade2 his3 leu2 lys2 trp1 ura3 fks1*::*HIS3 fks2*::*LYS2 ade3*::*fks1-1154*:*TRP1*	67, 68
	YOC5624	*KRE6*(*F552I*) *SKN1*	As BY4741 *kre6*::*URA3*:*KRE6*(*F552I*):*kanMX4*	This study
	YOC5626	*KRE6 skn1Δ*	As BY4741 *skn1Δ*::*kanMX4*	This study
	YOC5627	*kre6Δ SKN1*	As BY4741 *kre6Δ*::*kanMX4*	This study
	YOC5628	*KRE6 SKN1*(*F604I*)	As BY4741 *skn1*::*SKN1*(*F604I*):*HIS3*:*kanMX4*	This study
	YOC5629	*kre6Δ SKN1*(*F604I*)	As BY4741 *skn1*::*LEU2*::*SKN1*(*F604I*):*HIS3 kre6*::*kanMX4*	This study
	YOC5630	*KRE6*(*F552I*) *skn1Δ*	As BY4741 *skn1*::*LEU2 kre6*::*KRE6*(*F552I*):*natMX6*:*kanMX4*	This study
	YOC5631	*KRE6*(*F552I*) *SKN1*(*F604I*)	As BY4741 *skn1*::*SKN1*(*F604I*):*HIS3*:*LEU2 kre6*::*KRE6*(*F552I*):*natMX6*:*kanMX4*	This study
C. albicans	YOC5265	ATCC 24433		NBRP^*c*^
C. krusei	YOC5266	ATCC 6258		NBRP
C. tropicalis	YOC5267	ATCC 750		NBRP
C. parapsilosis	YOC5268	ATCC 22019		NBRP

a Yeast community used bold letter "**a**" as a meaning of mating type.

b *Kl, Kluyveromyces lactis; Sp, Schizosaccharomyces pombe*.

c National BioResource Project (NBRP) (64).

### Media.

Yeast cells were grown at 25°C in yeast extract-peptone-dextrose (YPD) rich medium containing 1% Bacto yeast extract (BD Biosciences, San Jose, CA), 2% Bacto peptone (BD Biosciences), and 2% glucose (Fujifilm Wako Pure Chemical Corporation, Osaka, Japan). For K1 killer sensitivity assays, the pH of YPD was decreased by adding a 1/10 volume of citrate-phosphate buffer (1.0 M citric acid, 1.62 M K_2_HPO_4_, pH 4.5). For drug sensitivity assays, RPMI 1640 (Fujifilm Wako Pure Chemical Corporation) was adjusted to pH 6.9 by adding 165 mM MOPS (morpholinepropanesulfonic acid). B. cinerea was grown at 25°C on potato dextrose agar (399-01841; Fujifilm Wako Pure Chemical Corporation) containing 0.4% potato extract, 2% glucose, and 1.5% agar. Pyricularia oryzae was grown at 25°C on oatmeal agar containing 5% oatmeal, 1% glucose, and 2% agar.

### Drugs.

Jervine (J0009; Tokyo Chemical Industry, Tokyo, Japan), FLC (F0677; Tokyo Chemical Industry), D75 (STOCK2S-79946; InterBioScreen, Chernogolovka, Russia), and 2,4,5,6-tetrachloroisophthalonitrile (Daconil 1000; Kumiai Chemical Industry, Tokyo, Japan) were purchased. EB was a gift from O. Kondo at Chugai Pharmaceutical. All drugs were dissolved in dimethyl sulfoxide (DMSO) and prepared as 100-fold-concentrated stocks.

### Morphological analysis.

Logarithmic-phase wild-type cells were fixed and stained with fluorescein isothiocyanate-concanavalin A (FITC-ConA) for mannoprotein, rhodamine-phalloidin for actin, and DAPI (4,6-diamidino-2-phenylindole) for nuclear DNA. Images were acquired at room temperature using a fluorescence microscope (Axioplan 2; Carl Zeiss AG, Oberkochen, Germany). A cooled charge-coupled device camera (CoolSNAP HQ; Roper Scientific Photometrics, Tucson, AZ, USA) was used for image capture. Yeast cell image analysis was performed using CalMorph software (version 1.2) as described previously (27). CalMorph automatically characterizes each yeast cell by calculating 501 morphologic parameters based on data from more than 200 cells. Morphological data for 4,718 nonessential-gene deletion mutants, the wild type (126 replicates), and cells treated with EB, TN, and NZ were obtained previously (27, 56, 57). Data were analyzed using R (http://www.r-project.org/). High-content image profiling, including the Pearson product-moment correlation analysis, was described previously (57).

### Chemical-genomics analysis.

Chemical-genomics profiling using a pool of 310 deletion mutant strains was performed as described previously (20). Briefly, pooled cultures were treated with the indicated concentrations of jervine (25, 12.5, and 6.25 μg/ml) and D75 (12.5, 6.25, and 3.13 μM) and grown for 48 h at 30°C. Purification of genomic DNA from harvested cells, PCR amplification of barcodes using multiplex primers, and gel purification of barcodes were carried out as described previously (20). Barcodes were sequenced on an Illumina MiSeq using MiSeq reagent kit version 3 (150 cycles; Illumina, Inc., San Diego, CA, USA). The barcode counts detected for each deletion mutant were quantified using BEAN-counter software to generate fitness-based chemical-genetic interaction scores (58). To compare multiple chemical-genomic profiles, we employed chemical-genomic profiles for jervine (12.5 μg/ml) and D75 (6.25 μM) as representatives (Table S1). The chemical-genomics profiles for the pseudojervine-related compounds (NP157, NP293, NP329, and NP413) and control compounds (TN, calcofluor white, and micafungin) were reported previously (20).

### β-1,3-Glucan staining.

β-1,3-Glucan staining was performed as described previously (19, 59). Yeast cells were cultured overnight at 25°C in YPD medium to ∼1 × 10^7^ cells/ml. The cells were washed in phosphate-buffered saline (PBS) and added to 5 mg/ml aniline blue. The signal intensity of aniline blue was quantified using ImageJ software version 1.49v. We binarized the image manually by the default method (Image → Adjust → Threshold color). Furthermore, a 1-pixel hole was filled (Process → Binary → Close) and a small area considered noise was deleted (Process → Binary → Open). The particle areas of cell were added to ROI Manager (Analyze → Analyze particles), and the mean fluorescence intensity of particle areas was measured using ROI Manager (Measure).

### Uptake of [^14^C]glucose into the cell wall.

The wild-type strain was cultured overnight at 25°C in YPD medium to ∼1 × 10^7^ cells/ml and adjusted to 1 × 10^7^ cells/ml in low-glucose YPD containing jervine (0, 2.5, 5.0, and 10.0 μg/ml) and 0.624 μCi [^14^C]glucose (ARC0122; American Radiolabeled Chemicals, Maryland Heights, Mo, USA). Cells were cultured at 25°C for 2 h, and β-1,6-glucan, β-1,3-glucan, and chitin fractions were prepared using a slightly modified protocol of Kitamura et al. (18). Five hundred microliters of 10% trichloroacetic acid (TCA) was added, and the culture was incubated on ice for 10 min. After centrifugation at 15,000 × *g* for 1 min, samples were washed twice with distilled water (DW). The pellet was suspended in 500 μl of 1 N NaOH and incubated at 75°C for 1 h. The mixture was centrifuged at 15,000 × *g* for 3 min, and the supernatant was discarded. After washing the cells with Tris buffer (10 mM Tris-HCl, pH 7.5), pellets were added to 100 μl of Zymolyase buffer (5 mg/ml Zymolyase 100 T, 10 mM Tris-HCl) and incubated at 37°C for 20 h. The mixture was centrifuged at 15,000 × *g* for 15 min, and the supernatant was loaded on a separation filter (UFC 501096; Merck Millipore, Burlington, MA, USA). Tris buffer was added to the pellet, and the pellet was centrifuged at 5,000 × *g* for 15 min. The supernatant was placed on a β-1,6-glucan separation filter. The pellet was suspended in 100 μl of Tris buffer to prepare a chitin fraction. The separation filter was centrifuged at 14,000 × *g* for 30 min, and the filtration fraction was the β-1,3-glucan fraction; the concentrated fraction was the β-1,6-glucan fraction. Five milliliters of scintillation cocktail (6013329; PerkinElmer, Waltham, MA, USA) and three fractions were added to a plastic vial and assayed using a scintillation counter (LSC-6100; Hitachi Aloka, Tokyo, Japan) for 3 min.

### K1 killer toxin susceptibility test.

The test strains (*his3Δ* and *kre6Δ* mutants) were cultured in YPD medium for 10 h at 30°C and diluted with YPD to an optical density at 600 nm (OD_600_) of 0.01 (4 × 10^5^ cells/ml). Cells (620 μl) were put on a low-pH YPD plate (pH 4.5) and dried. The K1 killer toxin-producing strain (S. cerevisiae NCYC 232) was cultured overnight at 20°C in YPD to an OD_600_ of 1.4, and cells (5 μl) were spotted on a plate. After incubation for 2 days at 25°C, the growth inhibition circle was quantified using ImageJ software version 1.49v. We binarized the image manually by the default method (Image → Adjust → Threshold color). A 1-pixel hole was filled (Process → Binary → Close), and a small area considered noise was deleted (Process → Binary → Open). The straight distance (from the end of the colony of NCYC 232 to the test strain) of the growth inhibition circles was manually drawn by ‘Straight.’ The straight distance was measured at four places per sample using ‘Measure’ in ROI Manager. A distance of 0 pixels was assumed to be 84.7 μm because 1 pixel = 84.7 μm.

### Quantification of β-1,6-glucan.

Wild-type and mutant S. cerevisiae strains were grown in YPD at 25°C with shaking at 200 rpm to 1 × 10^7^ cells/ml. The samples were centrifuged at 15,000 × *g* for 3 min, and the supernatant was discarded. The pellet was washed, suspended in PBS to adjust it to 1 × 10^6^ cells/ml, and then autoclaved for 20 min. After centrifugation at 15,000 × *g* for 1 min, the pellet was further extracted. The β-1,6-glucan was extracted from the pellet using a slightly modified version of the protocol of Kitamura et al. (18). First, 500 μl of 10% TCA was added to the culture, which was incubated on ice for 10 min. After centrifugation at 15,000 × *g* for 3 min, the samples were washed twice with DW. The pellet was suspended in 500 μl of 1 N NaOH and incubated at 75°C for 1 h. The solution was mixed with 500 μl of 1 M HCl and Tris buffer (10 mM Tris-HCl, pH 7). After centrifugation at 15,000 × *g* for 1 min, the supernatant was stored on ice. The total amounts of β-1,6-glucan were measured according to the method of Yamanaka et al. (32). Briefly, a 96-well white plate was coated with Neg1-E321Q-His (2 μg/ml), followed by overnight incubation at 4°C. The plate was washed with PBS containing 0.05% Tween 20 (PBST) and incubated for 1 h with PBST plus 1% bovine serum albumin (BPBST). After washing, the diluted specimen and standard β-1,6-glucan (pustulan; InvivoGen, San Diego, CA, USA) were added to the plate and incubated for 1 h at room temperature. Biotinized Neg1-E321Q-His (2 μg/ml) in PBS containing BPBST was added to the washed plate, which was incubated for 1 h. The plate was then washed and treated with streptavidin-horseradish peroxidase (HRP) (R&D Systems, MN, USA) in BPBST for 20 min. After removing the unbound enzyme, the peroxidase substrate (SuperSignal ELISA Femto substrate; Thermo Fisher Scientific, Waltham, MA, USA) was added, and luminescence signals were measured using a microplate reader (GloMax; Promega, Madison, WI, USA).

### Antifungal susceptibility test using S. cerevisiae mutants.

Saccharomyces cerevisiae wild-type and mutant cells were grown in YPD at 25°C or 30°C with shaking at 200 rpm overnight to logarithmic phase (1 × 10^7^ to 5 × 10^7^ cells/ml). Overnight cultures were diluted with YPD, inoculated into YPD containing 3% DMSO (with and without drugs) to 1 × 10^5^ to 5 × 10^5^ cells/ml, and incubated at 30°C in a static incubator. Jervine and D75 concentrations ranged from 0 to 128 μg/ml and 0 to 256 μg/ml, respectively. After 18 h of incubation in 96-well flat-bottom microtiter plates (Corning, Corning, NY, USA), the cell suspension was stirred with a Titramax 1000 rotator (Heidolph, Schwalbach, Germany). The OD_600_ was measured using a SpectraMax plus 384 plate reader (Molecular Devices, San Jose, CA, USA). The IC_50_ was estimated using the 4-parameter logistic equation in the drc package in R (60). To test whether two pairs of IC_50_s were statistically different, dose-response curves were generated according to Markov chain Monte Carlo methods with the rstan package (https://mc-stan.org/users/interfaces/rstan) and the 4-parameter log-logistic equation was reparametrized using the drc package in R (60). Next, we employed a likelihood ratio test between the full model and null model. The full model and null model assumed differences among all conditions in all four parameters of the log-logistic equation and differences except for the IC_50_ between the conditions, respectively. The *P* value was calculated from the chi-squared distribution after Bonferroni correction.

### Antifungal susceptibility testing in *Candida* species.

The susceptibility test for each fungal strain was measured by the Clinical and Laboratory Standards Institute microdilution method (CLSI M60 [61]). Saccharomyces cerevisiae wild-type and *Candida* (C. albicans, C. tropicalis, C. krusei, and C. parapsilosis) cells were grown on Sabouraud glucose agar at 30°C or 35°C for 24 to 48 h. Cells were washed with saline and diluted in RPMI 1640 to 2.5 × 10^3^ cells/ml. Diluted cells and 1% DMSO (with/without drugs) were added to 96-well round-bottom microplates and incubated at 25°C or 30°C in a static incubator. Jervine concentrations ranged from 0 to 64 μg/ml. Saccharomyces cerevisiae cells were grown at 30°C because they did not grow at 35°C. After 24 h of incubation, cell growth was assessed visually as follows: MIC_90_, optically clear; MIC_50_, prominent decrease in turbidity.

### Growth inhibition assay of phytopathogenic fungi.

Botrytis cinerea spores were harvested on potato dextrose agar and filtered through cloth. The spore concentration was determined, and the suspension diluted to 2 × 10^4^ spores/ml in DW. Puccinia recondita spores were harvested on wheat and stirred with 2,500-fold-diluted Tween 20. The spore concentration was determined, and the suspension was diluted to 6 × 10^4^ spores/ml. Pyricularia oryzae spores were harvested on oatmeal agar. The spore concentration was determined, and the suspension diluted to 2 × 10^4^ spores/ml in DW. Fifty microliters of diluted spores with 1% DMSO (with/without drugs) was added to 96-well flat-bottom microplates and incubated at 25°C for 48 h, and spore germination was evaluated under an optical microscope.

### Checkerboard assay.

Synergy was tested by the checkerboard method, a two-dimensional array of serial concentrations of test compounds, which is frequently used to assess combinations of antifungal agents *in vitro* (62). The tested dilutions were selected based on the MICs of each substance. Each fungal strain was exposed to various concentrations of jervine (0 to 64 μg/ml) in combination with fluconazole (0 to 256 μg/ml) or echinocandin B (0 to 64 μg/ml). The checkerboard test was used to calculate the FIC index (62) according to the following formulas: FIC_A_ = MIC_A+B_/MIC_A_, FIC_B_ = MIC_B+A_/MIC_B_, and FIC index = FIC_A_ + FIC_B_. The MIC_A+B_ value represents the MIC of compound A in the presence of compound B. FIC index values were interpreted as follows (63): synergy was shown by an FIC index of ≤0.5, antagonism by an FIC index of >4.0, and no interaction by an FIC index of >0.5 to 4.0. The test was performed in 96-well microtiter plates containing YPD supplemented with drugs in serial concentrations. Fungal suspensions were inoculated to a cell density of 5 × 10^5^ cells/ml. Plates were read after incubation for 18 h at 25°C. Each test was performed in triplicate.

### General synthesis procedures.

All reagents were of the highest commercial grade and applied directly unless otherwise stated. All reactions were performed under a nitrogen or argon atmosphere unless otherwise stated. Tetrahydrofuran (THF), toluene, hexane, dichloromethane, and ethyl acetate were purchased from Kishida Chemical (Osaka, Japan). Column chromatography was performed with silica gel (Wakogel 60N; Fujifilm Wako Pure Chemicals, Osaka, Japan). Analytical thin-layer chromatography (TLC) was performed with glass TLC plates (silica gel 70 F_254_ Plate-Wako; Fujifilm Wako Pure Chemicals, Osaka, Japan). The plates were visualized with UV light and phosphomolybdic acid and subsequent heating. Nuclear magnetic resonance (NMR) spectra were recorded using the ECX-400 instrument (JEOL, Tokyo, Japan). Chemical shift values are reported in ppm (δ) downfield from tetramethylsilane (0 ppm) with reference to an internal residual solvent [^1^H NMR, CHCl_3_ (7.26)]. The coupling constants (*J*) are reported in Hertz (Hz). The following abbreviations are used to designate the multiplicities: s, singlet; d, doublet; t, triplet; m, multiplet; and br, broad or combination peaks.

### Synthesis of cyclopamine-*N*-Boc.

Di-*tert*-butyl dicarbonate (Boc; 5.3 mg, 0.0243 mmol) was added to a solution of cyclopamine (10 mg, 0.0243 mmol) in THF/H_2_O/toluene (1:1:2, 10 ml) at room temperature and stirred at 90°C overnight. The reaction mixture was cooled to room temperature and concentrated under reduced pressure *in vacuo* to remove all solvents. This crude compound was purified by silica-gel column chromatography (2:1 ratio of hexane and ethyl acetate). The fractions containing the desired compounds were combined and concentrated under reduced pressure *in vacuo* to yield cyclopamine-*N*-Boc as a colorless solid (12.7 mg, <100%); *R_f_* = 0.36 (hexane:ethyl acetate =2:1); ^1^H NMR (400 MHz, CDCl_3_): 5.38 (brs, 1H), 3.56 to 3.51 (m, 3H), 3.15 (dd, *J *= 8.0, 4.0 Hz, 1H), 2.91 (dd, *J *= 16.0, 8.0 Hz, 1H), 2.80 to 2.95 (m, 1H), 2.40 to 2.35 (m, 1H), 2.30 to 2.10 (m, 6H), 1.87 to 1.63 (m, 6H), 1.69 (s, 3H), 1.60 to 1.52 (m, 2H), 1.48 (s, 9H), 1.49 to 1.01 (m, 6H), 1.00 (d, *J *= 8.0 Hz, 3H), 0.94 (s, 3H), 0.93 (d, *J *= 8.0 Hz, 3H).

### Synthesis of cyclopamine-*O*-Ac.

Acetic anhydride (0.15 ml) and diisopropylethylamine (0.23 ml) were added to a mixture of cyclopamine-*N*-Boc (5.6 mg [0.0109 mmol]) and dichloromethane (1 ml). The reaction mixture was stirred at 50°C for 5 h and cooled to room temperature; saturated aqueous NH_4_Cl (1 ml) was then added. The mixture was washed twice with ethyl acetate (5 ml), dried over sodium sulfate, filtered, and concentrated under reduced pressure. The crude compound was purified by silica gel column chromatography (4:1 ratio of hexane and ethyl acetate). The fractions containing the desired compounds were combined and concentrated under reduced pressure *in vacuo*, to yield cyclopamine-*N*-Boc-*O*-Ac as a colorless solid: (3.1 mg, brsm 60% yield); *R_f_* = 0.65 (hexane/ethyl acetate =2:1); ^1^H NMR (400 MHz, CDCl_3_): 5.32 (brs, 1H), 4.52 to 4.50 (m, 1H), 3.50 to 3.08 (m, 2H), 3.07 (dd, *J *= 8.0, 8.0 Hz, 1H), 2.84 (dd, *J *= 16.0, 8.0 Hz 1H), 2.56 to 2.54 (m, 1H), 2.35 to 2.05 (m, 6H), 1.97 (s, 3H), 1.85 to 1.60 (m, 6H), 1.62 (s, 3H), 1.55 to 1.45 (m, 2H), 1.45 (s, 9H), 1.45 to 1.10 (m, 6H), 0.93 (d, *J *= 8.0 Hz, 3H), 0.88 (s, 3H), 0.86 (d, *J *= 8.0 Hz, 3H).

Trifluoroacetic acid (0.03 ml) was added dropwise to a mixture of cyclopamine-*N*-Boc-*O*-Ac (3.1 mg [5.6 μmol]) and dichloromethane (1 ml) at 0°C. The reaction mixture was stirred for 10 min and concentrated under reduced pressure to remove trifluoroacetic acid. The crude compound was purified by silica-gel column chromatography (ratio of dichloromethane/methanol of 30:1 to 10:1). Fractions containing the desired compounds were combined and concentrated under reduced pressure *in vacuo*, to yield cyclopamine-*O*-Ac as a colorless solid: (0.87 mg, 34% yield); *R_f_* = 0.50 (dichloromethane/methanol = 5:1); ^1^H NMR (400 MHz, CDCl_3_): 5.38 (brs, 1H), 4.53 to 4.51 (m, 1H), 3.28 to 3.15 (td, *J *= 8.0, 4.0 Hz, 1H), 3.07 (dd, *J *= 8.0, 8.0 Hz, 1H), 2.66 (dd, *J *= 8.0, 8.0 Hz, 1H), 2.44 to 2.10 (m, 8H), 2.03 (s, 3H), 1.92 to 1.15 (m, 18H including 1.64 [s, 3H]), 0.95 (d, *J *= 8.0 Hz, 3H), 0.94 (s, 3H), 0.93 (d, *J *= 8.0 Hz, 3H).

## References

[1] Fisher MC, Henk DA, Briggs CJ, Brownstein JS, Madoff LC, McCraw SL, Gurr SJ. 2012. Emerging fungal threats to animal, plant and ecosystem health. Nature 484:186–194. doi:10.1038/nature10947. 22498624 PMC3821985

[2] Pfaller MA, Diekema DJ. 2007. Epidemiology of invasive candidiasis: a persistent public health problem. Clin Microbiol Rev 20:133–163. doi:10.1128/CMR.00029-06. 17223626 PMC1797637

[3] Strange RN, Scott PR. 2005. Plant disease: a threat to global food security. Annu Rev Phytopathol 43:83–116. doi:10.1146/annurev.phyto.43.113004.133839. 16078878

[4] Dean R, Van Kan JAL, Pretorius ZA, Hammond-Kosack KE, Di Pietro A, Spanu PD, Rudd JJ, Dickman M, Kahmann R, Ellis J, Foster GD. 2012. The top 10 fungal pathogens in molecular plant pathology. Mol Plant Pathol 13:414–430. doi:10.1111/j.1364-3703.2011.00783.x. 22471698 PMC6638784

[5] Soanes DM, Richards TA, Talbot NJ. 2007. Insights from sequencing fungal and oomycete genomes: what can we learn about plant disease and the evolution of pathogenicity? Plant Cell 19:3318–3326. doi:10.1105/tpc.107.056663. 18024565 PMC2174898

[6] Chen SN, Luo CX, Hu MJ, Schnabel G. 2016. Fitness and competitive ability of botrytis cinerea isolates with resistance to multiple chemical classes of fungicides. Phytopathology 106:997–1005. doi:10.1094/PHYTO-02-16-0061-R. 27161219

[7] Fisher MC, Hawkins NJ, Sanglard D, Gurr SJ. 2018. Worldwide emergence of resistance to antifungal drugs challenges human health and food security. Science 360:739–742. doi:10.1126/science.aap7999. 29773744

[8] Geddes-McAlister J, Shapiro RS. 2019. New pathogens, new tricks: emerging, drug-resistant fungal pathogens and future prospects for antifungal therapeutics. Ann N Y Acad Sci 1435:57–78. doi:10.1111/nyas.13739. 29762860

[9] Pristov KE, Ghannoum MA. 2019. Resistance of candida to azoles and echinocandins worldwide. Clin Microbiol Infect 25:792–798. doi:10.1016/j.cmi.2019.03.028. 30965100

[10] Cortés JCG, Curto MÁ, Carvalho VSD, Pérez P, Ribas JC. 2019. The fungal cell wall as a target for the development of new antifungal therapies. Biotechnol Adv 37:107352–107375. doi:10.1016/j.biotechadv.2019.02.008. 30797093

[11] Liu W, Yuan L, Wang S. 2020. Recent progress in the discovery of antifungal agents targeting the cell wall. J Med Chem 63:12429–12459. doi:10.1021/acs.jmedchem.0c00748. 32692166

[12] Debono M, Gordee RS. 1994. Antibiotics that inhibit fungal cell wall development. Annu Rev Microbiol 48:471–497. doi:10.1146/annurev.mi.48.100194.002351. 7826015

[13] Georgopapadakou NH, Tkacz JS. 1995. The fungal cell wall as a drug target. Trends Microbiol 3:98–104. doi:10.1016/s0966-842x(00)88890-3. 7773595

[14] Gow NAR, Latge J-P, Munro CA. 2017. The fungal cell wall: structure, biosynthesis, and function. Microbiol Spectr 5:5.3.01. doi:10.1128/microbiolspec.FUNK-0035-2016. PMC11687499 28513415

[15] Douglas CM, Marrinan JA, Li W, Kurtz MB. 1994. A Saccharomyces cerevisiae mutant with echinocandin-resistant 1,3-β-D-glucan synthase. J Bacteriol 176:5686–5696. doi:10.1128/jb.176.18.5686-5696.1994. 8083161 PMC196772

[16] Hüttel W. 2017. Structural diversity in echinocandin biosynthesis: the impact of oxidation steps and approaches toward an evolutionary explanation. Z Naturforsch C J Biosci 72:1–20. doi:10.1515/znc-2016-0156. 27705900

[17] Fungicide Resistance Action Committee. 2021. FRAC classification of fungicides: fungal control agents by cross resistance pattern and mode of action 2021. https://www.frac.info/docs/default-source/publications/frac-mode-of-action-poster/frac-moa-poster-2021.pdf?sfvrsn=a6f6499a_2. Accessed 1 July 2021.

[18] Kitamura A, Someya K, Hata M, Nakajima R, Takemura M. 2009. Discovery of a small-molecule inhibitor of β-1,6-glucan synthesis. Antimicrob Agents Chemother 53:670–677. doi:10.1128/AAC.00844-08. 19015325 PMC2630612

[19] Piotrowski JS, Okada H, Lu F, Li SC, Hinchman L, Ranjan A, Smith DL, Higbee AJ, Ulbrich A, Coon JJ, Deshpande R, Bukhman YV, McIlwain S, Ong IM, Myers CL, Boone C, Landick R, Ralph J, Kabbage M, Ohya Y. 2015. Plant-derived antifungal agent poacic acid targets β-1,3-glucan. Proc Natl Acad Sci USA 112:E1490–E1497. doi:10.1073/pnas.1410400112. 25775513 PMC4378397

[20] Piotrowski JS, Li SC, Deshpande R, Simpkins SW, Nelson J, Yashiroda Y, Barber JM, Safizadeh H, Wilson E, Okada H, Gebre AA, Kubo K, Torres NP, Leblanc MA, Andrusiak K, Okamoto R, Yoshimura M, Derango-Adem E, van Leeuwen J, Shirahige K, Baryshnikova A, Brown GW, Hirano H, Costanzo M, Andrews B, Ohya Y, Osada H, Yoshida M, Myers CL, Boone C. 2017. Functional annotation of chemical libraries across diverse biological processes. Nat Chem Biol 13:982–993. doi:10.1038/nchembio.2436. 28759014 PMC6056180

[21] Jacobs WA, Raig LC. 1943. The veratrine alkaloids. J Biol Chem 148:51–55. doi:10.1016/S0021-9258(18)72315-6.

[22] Chen JK, Taipale J, Cooper MK, Beachy PA. 2002. Inhibition of hedgehog signaling by direct binding of cyclopamine to smoothened. Genes Dev 16:2743–2748. doi:10.1101/gad.1025302. 12414725 PMC187469

[23] Liu M, Lu X, Zhang J, Zhao X, Zhang W, Lin X. 2019. Teratogenic jervine increases the activity of doxorubicin in MCF-7/ADR cells by inhibiting ABCB1. Biomed Pharmacother 117:109059. doi:10.1016/j.biopha.2019.109059. 31207578

[24] Qin YT, Jiang M, Tuerxung N, Wang H, Zhao F, Zhen Y, Hao J. 2020. Sonic hedgehog signaling pathway in myelodysplastic syndrome: abnormal activation and jervine intervention. Gene 754:144881. doi:10.1016/j.gene.2020.144881. 32526259

[25] Chen J, Wen B, Wang Y, Wu S, Zhang X, Gu Y, Wang Z, Wang J, Zhang W, Yong J. 2020. Jervine exhibits anticancer effects on nasopharyngeal carcinoma through promoting autophagic apoptosis via the blockage of hedgehog signaling. Biomed Pharmacother 132:110898. doi:10.1016/j.biopha.2020.110898. 33113432

[26] Keeler RF. 1970. Teratogenic compounds of *Veratrum californicum* (Durand) X. Cyclopia in rabbits produced by cyclopamine. Teratology 3:175–180. doi:10.1002/tera.1420030210. 4986632

[27] Ohya Y, Sese J, Yukawa M, Sano F, Nakatani Y, Saito TL, Saka A, Fukuda T, Ishihara S, Oka S, Suzuki G, Watanabe M, Hirata A, Ohtani M, Sawai H, Fraysse N, Latgé JP, François JM, Aebi M, Tanaka S, Muramatsu S, Araki H, Sonoike K, Nogami S, Morishita S. 2005. High-dimensional and large-scale phenotyping of yeast mutants. Proc Natl Acad Sci USA 102:19015–19020. doi:10.1073/pnas.0509436102. 16365294 PMC1316885

[28] Breinig F, Tipper DJ, Schmitt MJ. 2002. Kre1p, the plasma membrane receptor for the yeast K1 viral toxin. Cell 108:395–405. doi:10.1016/S0092-8674(02)00634-7. 11853673

[29] Fredericks LR, Lee MD, Crabtree AM, Boyer JM, Kizer EA, Taggart NT, Roslund CR, Hunter SS, Kennedy CB, Willmore CG, Tebbe NM, Harris JS, Brocke SN, Rowley PA. 2021. The species-specific acquisition and diversification of a K1-like family of killer toxins in budding yeasts of the Saccharomycotina. PLoS Genet 17:e1009341. doi:10.1371/journal.pgen.1009341. 33539346 PMC7888664

[30] Pagé N, Gérard-Vincent M, Ménard P, Beaulieu M, Azuma M, Dijkgraaf GJP, Li H, Marcoux J, Nguyen T, Dowse T, Sdicu AM, Bussey H. 2003. A *Saccharomyces cerevisiae* genome-wide mutant screen for altered sensitivity to K1 killer toxin. Genetics 163:875–894. doi:10.1093/genetics/163.3.875. 12663529 PMC1462477

[31] Roemer T, Delaney S, Bussey H. 1993. SKN1 and KRE6 define a pair of functional homologs encoding putative membrane proteins involved in beta-glucan synthesis. Mol Cell Biol 13:4039–4048. doi:10.1128/mcb.13.7.4039-4048.1993. 8321211 PMC359953

[32] Yamanaka D, Takatsu K, Kimura M, Swamydas M, Ohnishi H, Umeyama T, Oyama F, Lionakis MS, Ohno N. 2020. Development of a novel β-1,6-glucan-specific detection system using functionally-modified recombinant endo-β-1,6-glucanase. J Biol Chem 295:5362–5376. doi:10.1074/jbc.RA119.011851. 32132174 PMC7170528

[33] Ostrosky-Zeichner L. 2008. Combination antifungal therapy: a critical review of the evidence. Clin Microbiol Infect 14:65–70. doi:10.1111/j.1469-0691.2008.01983.x. 18430131

[34] Day JN, Chau TTH, Wolbers M, Mai PP, Dung NT, Mai NH, Phu NH, Nghia HD, Phong ND, Thai CQ, Thai LH, Chuong LV, Sinh DX, Duong VA, Hoang TN, Diep PT, Campbell JI, Sieu TPM, Baker SG, Chau NVV, Hien TT, Lalloo DG, Farrar JJ. 2013. Combination antifungal therapy for cryptococcal meningitis. N Engl J Med 368:1291–1302. doi:10.1056/NEJMoa1110404. 23550668 PMC3978204

[35] Goff RD, Thorson JS. 2012. Enhancement of cyclopamine via conjugation with nonmetabolic sugars. Org Lett 14:2454–2457. doi:10.1021/ol300703z. 22540932 PMC3442133

[36] Olive KP, Jacobetz MA, Davidson CJ, Gopinathan A, McIntyre D, Honess D, Madhu B, Goldgraben MA, Caldwell ME, Allard D, Frese KK, DeNicola G, Feig C, Combs C, Winter SP, Ireland-Zecchini H, Reichelt S, Howat WJ, Chang A, Dhara M, Wang L, Rückert F, Grützmann R, Pilarsky C, Izeradjene K, Hingorani SR, Huang P, Davies SE, Plunkett W, Egorin M, Hruban RH, Whitebread N, McGovern K, Adams J, Iacobuzio-Donahue C, Griffiths J, Tuveson DA. 2009. Inhibition of hedgehog signaling enhances delivery of chemotherapy in a mouse model of pancreatic cancer. Science 324:1457–1461. doi:10.1126/science.1171362. 19460966 PMC2998180

[37] Lin TL, Wang QH, Brown P, Peacock C, Merchant AA, Brennan S, Jones E, McGovern K, Watkins DN, Sakamoto KM, Matsui W. 2010. Self-renewal of acute lymphocytic leukemia cells is limited by the hedgehog pathway inhibitors cyclopamine and IPI-926. PLoS One 5:e15262. doi:10.1371/journal.pone.0015262. 21203400 PMC3011010

[38] Tremblay MR, Nevalainen M, Nair SJ, Porter JR, Castro AC, Behnke ML, Yu LC, Hagel M, White K, Faia K, Grenier L, Campbell MJ, Cushing J, Woodward CN, Hoyt J, Foley MA, Read MA, Sydor JR, Tong JK, Palombella VJ, McGovern K, Adams J. 2008. Semisynthetic cyclopamine analogues as potent and orally bioavailable hedgehog pathway antagonists. J Med Chem 51:6646–6649. doi:10.1021/jm8008508. 18842035

[39] Tremblay MR, Lescarbeau A, Grogan MJ, Tan E, Lin G, Austad BC, Yu LC, Behnke ML, Nair SJ, Hagel M, White K, Conley J, Manna JD, Alvarez-Diez TM, Hoyt J, Woodward CN, Sydor JR, Pink M, MacDougall J, Campbell MJ, Cushing J, Ferguson J, Curtis MS, McGovern K, Read MA, Palombella VJ, Adams J, Castro AC. 2009. Discovery of a potent and orally active hedgehog pathway antagonist (IPI-926). J Med Chem 52:4400–4418. doi:10.1021/jm900305z. 19522463

[40] Wright CRA, Luff AP. 1879. XLVI.—The alkaloïds of the veratrums. Part II. The alkaloïds of veratrum album. J Chem Soc 35:405–420. doi:10.1039/CT8793500405.

[41] Roemer T, Paravicini G, Payton MA, Bussey H. 1994. Characterization of the yeast (l,6)- β-glucan biosynthetic components, Kre6p and Sknlp, and genetic interactions between the PKC1 pathway and extracellular matrix assembly. J Cell Biol 127:567–579. doi:10.1083/jcb.127.2.567. 7929594 PMC2120205

[42] Han Q, Wang N, Yao G, Mu C, Wang Y, Sang J. 2019. Blocking β-1,6-glucan synthesis by deleting KRE6 and SKN1 attenuates the virulence of *Candida albicans*. Mol Microbiol 111:604–620. doi:10.1111/mmi.14176. 30507002

[43] Chhetri A, Loksztejn A, Nguyen H, Pianalto KM, Kim MJ, Hong J, Alspaugh JA, Yokoyama K. 2020. Length specificity and polymerization mechanism of (1,3)-β-d-glucan synthase in fungal cell wall biosynthesis. Biochem 59:682–693. doi:10.1021/acs.biochem.9b00896. 31899625 PMC7015794

[44] Perlin DS. 2007. Resistance to echinocandin-class antifungal drugs. Drug Resist Updat 10:121–130. doi:10.1016/j.drup.2007.04.002. 17569573 PMC2696280

[45] Johnson ME, Katiyar SK, Edlind TD. 2011. New Fks hot spot for acquired echinocandin resistance in *Saccharomyces cerevisiae* and its contribution to intrinsic resistance of Scedosporium species. Antimicrob Agents Chemother 55:3774–3781. doi:10.1128/AAC.01811-10. 21576441 PMC3147641

[46] Kitamura A, Higuchi S, Hata M, Kawakami K, Yoshida K, Namba K, Nakajima R. 2009. Effect of β-1,6-glucan inhibitors on the invasion process of *Candida albicans*: potential mechanism of their *in vivo* efficacy. Antimicrob Agents Chemother 53:3963–3971. doi:10.1128/AAC.00435-09. 19596881 PMC2737848

[47] Kitamura A, Someya K, Okumura R, Hata M, Takeshita H, Nakajima R. 2010. Antifungal and antibacterial susceptibility testing. Biol Pharm Bull 33:192–197. doi:10.1248/bpb.33.192. 20118539

[48] Chen SCA, Marriott D, Playford EG, Nguyen Q, Ellis D, Meyer W, Sorrell TC, Slavin M, Australian Candidaemia Study. 2009. Candidaemia with uncommon *Candida* species: predisposing factors, outcome, antifungal susceptibility, and implications for management. Clin Microbiol Infect 15:662–669. doi:10.1111/j.1469-0691.2009.02821.x. 19614718

[49] Pfaller MA, Diekema DJ. 2010. Epidemiology of invasive mycoses in North America. Crit Rev Microbiol 36:1–53. doi:10.3109/10408410903241444. 20088682

[50] Miceli MH, Díaz JA, Lee SA. 2011. Emerging opportunistic yeast infections. Lancet Infect Dis 11:142–151. doi:10.1016/S1473-3099(10)70218-8. 21272794

[51] Pfaller MA, Diekema DJ, Messer SA, Boyken L, Hollis RJ, Jones RN, Steele-Moore L, Denys G, Staley C, Dispersio JR, Saubolle M, Wilson ML, Overturf GD, Peterson LR, Schreckenberger PC, Doern GV. 2003. *In vitro* activities of voriconazole, posaconazole, and four licensed systemic antifungal agents against candida species infrequently isolated from blood. J Clin Microbiol 41:78–83. doi:10.1128/JCM.41.1.78-83.2003. 12517829 PMC149631

[52] Silva S, Negri M, Henriques M, Oliveira R, Williams DW, Azeredo J. 2012. *Candida glabrata*, *Candida parapsilosis* and *Candida tropicalis*: biology, epidemiology, pathogenicity and antifungal resistance. FEMS Microbiol Rev 36:288–305. doi:10.1111/j.1574-6976.2011.00278.x. 21569057

[53] Xiao M, Fan X, Chen SCA, Wang H, Sun ZY, Liao K, Chen SL, Yan Y, Kang M, Hu ZD, Chu YZ, Hu TS, Ni YX, Zou GL, Kong F, Xu YC. 2015. Antifungal susceptibilities of *Candida glabrata* species complex, *Candida krusei*, *Candida parapsilosis* species complex and *Candida tropicalis* causing invasive candidiasis in China: 3 year national surveillance. J Antimicrob Chemother 70:802–810. doi:10.1093/jac/dku460. 25473027

[54] Pfaller MA, Diekema DJ, Andes D, Arendrup MC, Brown SD, Lockhart SR, Motyl M, Perlin DS, CLSI Subcommittee for Antifungal Testing. 2011. Clinical breakpoints for the echinocandins and *Candida* revisited: integration of molecular, clinical, and microbiological data to arrive at species-specific interpretive criteria. Drug Resist Updat 14:164–176. doi:10.1016/j.drup.2011.01.004. 21353623

[55] Keeler RF, Binns W. 1968. Teratogenic compounds of *Veratrum californicum* (Durand). V. Comparison of cyclopian effects of steroidal alkaloids from the plant and structurally related compounds from other sources. Teratology 1:5–10. doi:10.1002/tera.1420010103. 5696817

[56] Ohnuki S, Oka S, Nogami S, Ohya Y. 2010. High-content, image-based screening for drug targets in yeast. PLoS One 5:e10177. doi:10.1371/journal.pone.0010177. 20418956 PMC2854693

[57] Okada H, Ohnuki S, Roncero C, Konopka JB, Ohya Y. 2014. Distinct roles of cell wall biogenesis in yeast morphogenesis as revealed by multivariate analysis of high-dimensional morphometric data. Mol Biol Cell 25:222–233. doi:10.1091/mbc.E13-07-0396. 24258022 PMC3890343

[58] Simpkins SW, Deshpande R, Nelson J, Li SC, Piotrowski JS, Ward HN, Yashiroda Y, Osada H, Yoshida M, Boone C, Myers CL. 2019. Using BEAN-counter to quantify genetic interactions from multiplexed barcode sequencing experiments. Nat Protoc 14:415–440. doi:10.1038/s41596-018-0099-1. 30635653 PMC6818255

[59] Okada H, Ohya Y. 2016. Fluorescent labeling of yeast cell wall components. Cold Spring Harb Protoc 2016:pdb.prot085241. doi:10.1101/pdb.prot085241. 27480714

[60] Ritz C, Baty F, Streibig JC, Gerhard D. 2015. Dose-response analysis using R. PLoS One 10:e0146021. doi:10.1371/journal.pone.0146021. 26717316 PMC4696819

[61] Clinical and Laboratory Standards Institute. 2020. Performance standards for antifungal susceptibility testing of yeasts, 2nd ed. CLSI M60. Clinical and Laboratory Standards Institute, Wayne, PA.

[62] Bidaud AL, Schwarz P, Herbreteau G, Dannaoui E. 2021. Techniques for the assessment of in vitro and in vivo antifungal combinations. J Fungi (Basel) 7:113. doi:10.3390/jof7020113. 33557026 PMC7913650

[63] Odds FC. 2003. Synergy, antagonism, and what the chequerboard puts between them. J Antimicrob Chemother 52:1. doi:10.1093/jac/dkg301. 12805255

[64] Clinical and Laboratory Standards Institute. 2002. Reference method for broth dilution antifungal susceptibility testing of yeasts; approved standard, 2nd ed. NCCLS M27-A2. National Committee for Clinical Laboratory Standards, Wayne, PA.

[65] Costanzo M, VanderSluis B, Koch EN, Baryshnikova A, Pons C, Tan G, Wang W, Usaj M, Hanchard J, Lee SD, Pelechano V, Styles EB, Billmann M, van Leeuwen J, van Dyk N, Lin ZY, Kuzmin E, Nelson J, Piotrowski JS, Srikumar T, Bahr S, Chen Y, Deshpande R, Kurat CF, Li SC, Li Z, Usaj MM, Okada H, Pascoe N, San Luis BJ, Sharifpoor S, Shuteriqi E, Simpkins SW, Snider J, Suresh HG, Tan Y, Zhu H, Malod-Dognin N, Janjic V, Przulj N, Troyanskaya OG, Stagljar I, Xia T, Ohya Y, Gingras AC, Raught B, Boutros M, Steinmetz LM, Moore CL, Rosebrock AP, Caudy AA, Myers CL, Andrews B, Boone C. 2016. A global genetic interaction network maps a wiring diagram of cellular function. Science 353:aaf1420. 27708008 10.1126/science.aaf1420 PMC5661885

[66] Nakamata K, Kurita T, Bhuiyan MS, Sato K, Noda Y, Yoda K. 2007. KEG1/YFR042w encodes a novel Kre6-binding endoplasmic reticulum membrane protein responsible for beta-1,6-glucan synthesis in *Saccharomyces cerevisiae*. J Biol Chem 282:34315–24. 17893149 10.1074/jbc.M706486200

[67] Sekiya-Kawasaki M, Abe M, Saka A, Watanabe D, Kono K, Minemura-Asakawa M, Ishihara S, Watanabe T, Ohya Y. 2002. Dissection of upstream regulatory components of the Rho1p effector, 1,3-beta-glucan synthase, in Saccharomyces cerevisiae. Genetics 162:663–76. 12399379 10.1093/genetics/162.2.663 PMC1462274

[68] Okada H, Abe M, Asakawa-Minemura M, Hirata A, Qadota H, Morishita K, Ohnuki S, Nogami S, Ohya Y. 2010. Multiple functional domains of the yeast l,3-beta-glucan synthase subunit Fks1p revealed by quantitative phenotypic analysis of temperature-sensitive mutants. Genetics 184:1013–24. 20124029 10.1534/genetics.109.109892 PMC2865904

