# Supplementary material for: Jerveratrum-Type Steroidal Alkaloids Inhibit β-1,6-Glucan Biosynthesis in Fungal Cell Walls
Source: Microbiol Spectr. 2022 Jan 12;10(1):e00873-21. doi: 10.1128/spectrum.00873-21 (PMC8754110; doi:10.1128/spectrum.00873-21)
Supplement: SUPPLEMENTAL FILE 1 — Supplemental material. Download SPECTRUM00873-21_Supp_1_seq16.pdf, PDF file, 0.4 MB [file spectrum00873-21_supp_1_seq16.pdf]

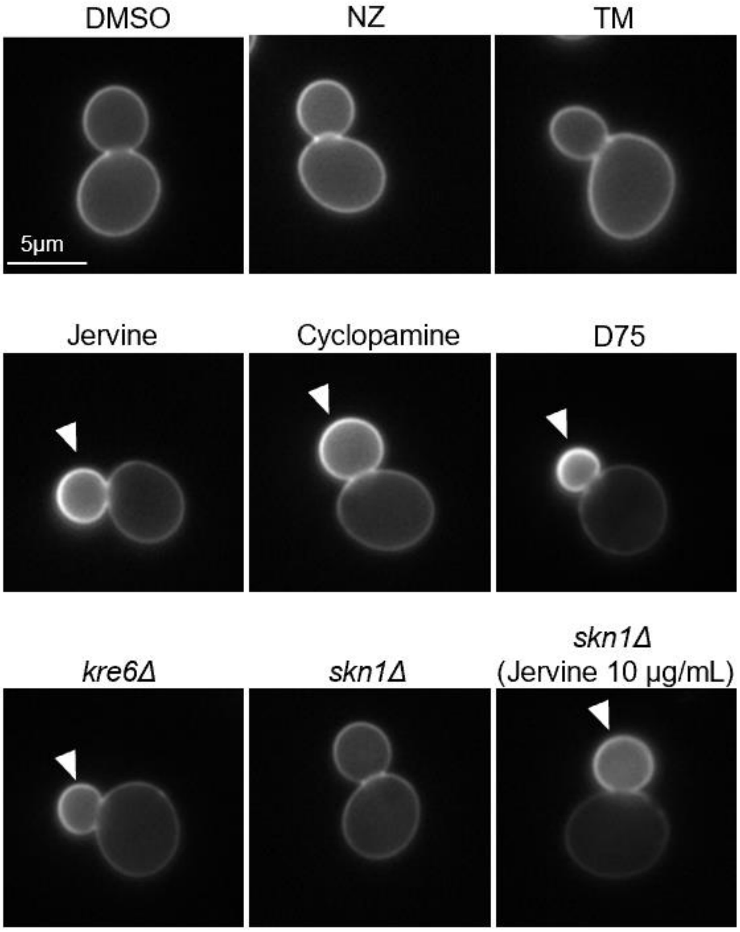

Supplementary Fig. S1. Localization of β-1,3-glucan after treatment with antifungal agents. Wild-type (*his3*Δ) cells were cultured with 1% DMSO, 198 μg/mL of nikkomycin Z (NZ), 0.1 μg/mL of tunicamycin (TM), 10 μg/mL of jervine, 10 μg/mL of cyclopamine, or 10 μg/mL of D75-4590 (D75) for 2 hours, and stained with aniline blue. *kre6Δ* and *skn1Δ* cells were cultured with 1% DMSO and stained with aniline blue. The arrowhead indicates increased β-1,3-glucan.

A

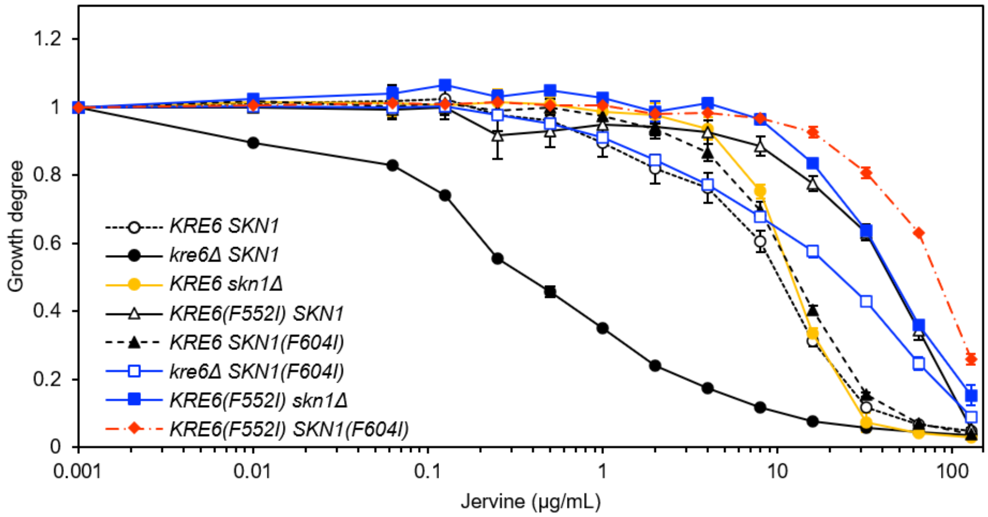

B

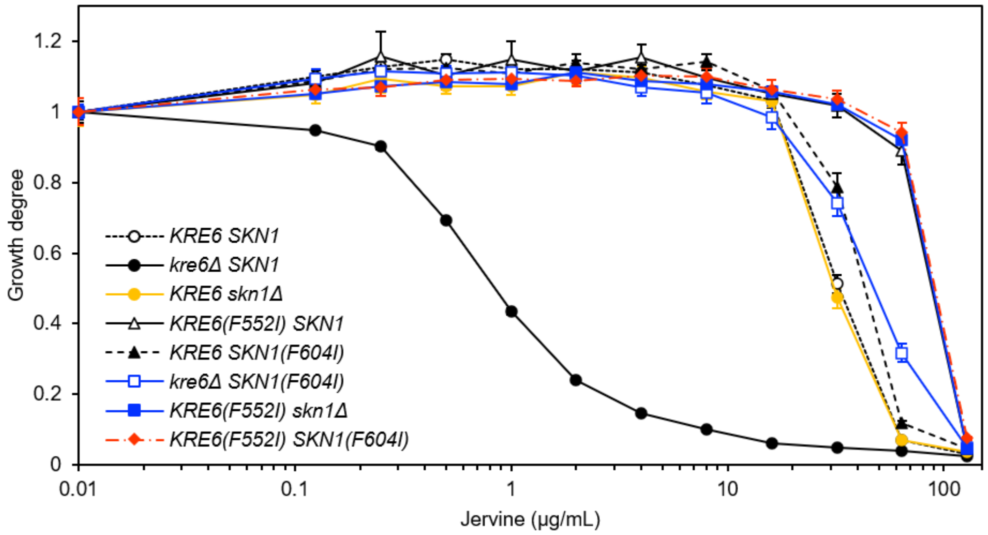

Supplementary Fig. S2. Jervine sensitivity of *KRE6(F552I)*, *SKN1(F604I)*, and other strains at 25°C (A) and at 30°C (B). Experiments were performed as in Figure 6A.

A

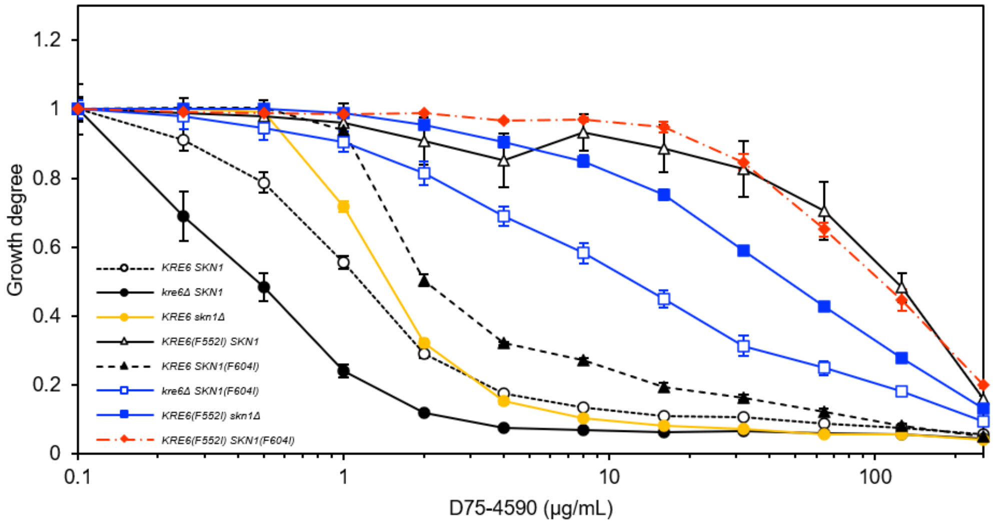

B

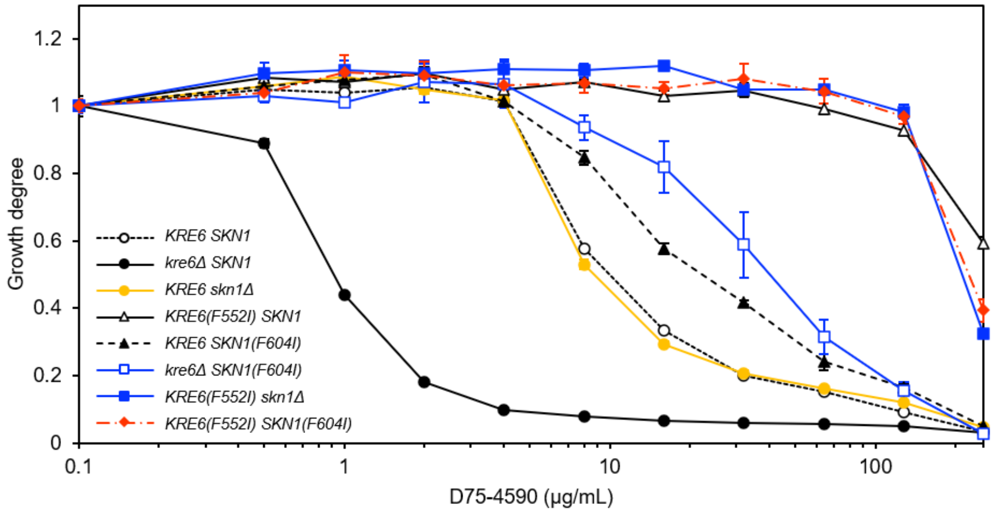

Supplementary Fig. S3. D75 sensitivity of *KRE6(F552I)*, *SKN1(F604I)*, and other strains at 25°C (A) and at 30°C (B). Experiments were performed as in Figure 6A. D75: D75-4590.

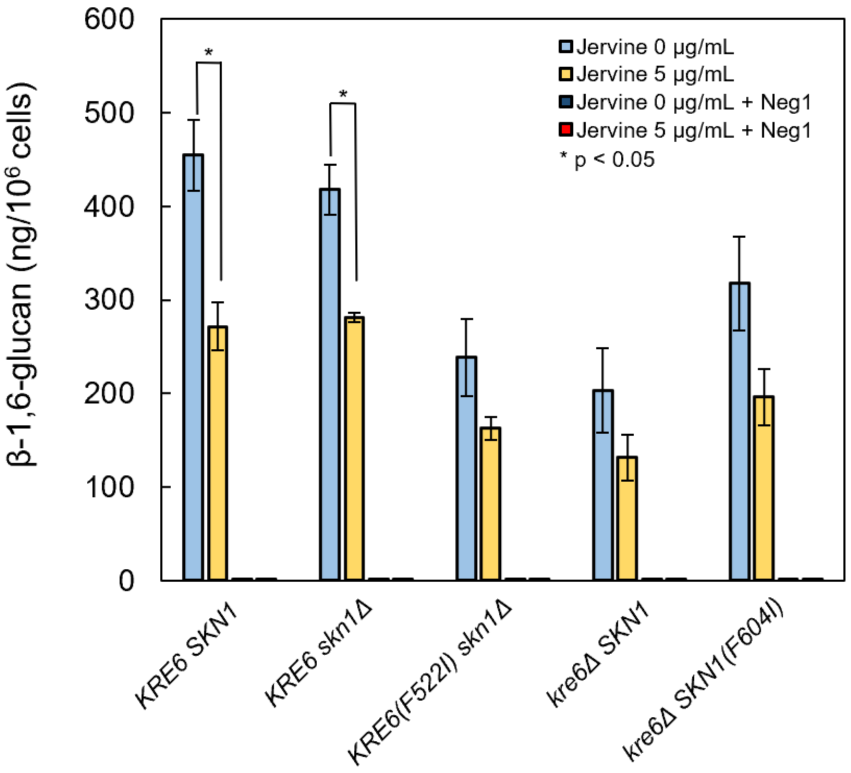

Supplementary Fig. S4. β-1,6-Glucan levels in yeast strains after treatment with jervine.

Yeast mutant strains were incubated in YPD without or with jervine (5 μg/mL) at 25°C for 4 hours. Cell proliferation was quantified by measuring OD_{600}. $OD_{600} = 1$ in the control condition ($n = 3$). Specimens were examined following treatment with modifying recombinant Neg1, a *Neurospora crassa* endo-β-1,6-glucanase. The amount of glucan per cell was calculated using pustulan as a standard. Significant differences between jervine treatment and non-treatment conditions are indicated with an asterisk (* $p < 0.05$ after Bonferroni correction, *t*-test).
